# Redox Regulation of Plant–Root-Knot Nematode Interactions: From ROS-Mediated Immunity to Sustainable Resistance

**DOI:** 10.3390/antiox15070853

**Published:** 2026-07-06

**Authors:** Jung-Wook Yang, Ho Soo Kim, Yun-Hee Kim

**Affiliations:** 1 Crop Environment Research Division, National Institute of Crop and Food Science, Wanju-gun 55365, Republic of Korea; hg369732@korea.kr; 2 Plant Systems Engineering Research Center, Korea Research Institute of Bioscience and Biotechnology (KRIBB), 125 Gwahak-ro, Daejeon 34141, Republic of Korea; 3 Department of Biology Education, IALS, Gyeongsang National University, Jinju 52828, Republic of Korea

**Keywords:** root-knot nematodes, reactive oxygen species, redox homeostasis, plant immunity, hydrogen peroxide

## Abstract

Root-knot nematodes (RKNs; *Meloidogyne* spp.) are among the most destructive plant parasites, causing severe yield losses in diverse crops. Reactive oxygen species (ROS), particularly superoxide radicals (O_2_•^−^) and hydrogen peroxide (H_2_O_2_), are central regulators of plant–RKN interactions. This review synthesizes current molecular, biochemical, genetic, transcriptomic, and translational evidence showing that the outcome of infection is determined by the spatiotemporal regulation of H_2_O_2_ rather than by ROS abundance alone. In resistant interactions, nematode perception activates PTI-associated signaling through selected cell-surface receptor complexes, including some BAK1/SERK3-associated pathways, together with BIK1, Ca^2+^ signaling, and RBOHD/F, generating a sustained oxidative activity associated with salicylic acid-dependent immune signaling and reduced H_2_O_2_-scavenging capacity and coupled to hypersensitive response, lignin and callose deposition, and feeding site restriction. In susceptible interactions, RKNs deploy ROS-targeting effectors such as Mi-CRT, MjTTL5, CATLe, Mj-NEROSs, and CMII to suppress ROS production, enhance antioxidant scavenging, or weaken SA-dependent defense. Evidence from a cyst-nematode system suggests that RBOH-derived ROS can restrict excessive cell death around syncytia; whether an analogous lower-redox requirement exists in RKN-induced giant cells remains unresolved. Finally, redox-based strategies, including CRISPR/Cas editing, host-induced gene silencing, chemical priming, and biocontrol, are discussed as promising approaches for durable and sustainable nematode resistance.

## 1. Introduction

Plant-parasitic nematodes are among the most destructive constraints on global agriculture, with estimated annual crop losses exceeding USD 157 billion across crop systems [1,2]. Among them, root-knot nematodes (RKNs) of the genus *Meloidogyne* are particularly damaging obligate sedentary endoparasites. The four major species—*M. incognita*, *M. javanica*, *M. arenaria*, and *M. hapla*—collectively parasitize more than 2000 plant species and are estimated to cause yield losses approaching or exceeding USD 100 billion annually [3]. Their broad host range, high reproductive potential, wide geographic distribution, and capacity to overcome or evade chemical and genetic control strategies make RKNs a persistent threat to sustainable crop production. Economically important crops are all severely affected by RKN infection [1,2,4].

The RKN infection cycle begins when infective second-stage juveniles (J2s) hatch from eggs in the rhizosphere and migrate toward host roots in response to root exudates and chemical gradients. J2s penetrate the elongation zone near the root tip and migrate intercellularly toward the vascular cylinder. Once they reach suitable feeding sites, they insert their stylets into selected parenchyma, pericycle, or endodermal cells and deliver esophageal gland secretions that reprogram host cell development [5]. These secretions induce repeated nuclear divisions without cytokinesis, resulting in multinucleate, hypertrophied, and metabolically active giant cells, also referred to as nurse or feeding cells [6,7]. Giant cells function as permanent nutrient sinks that supply solutes, amino acids, and other metabolites required for nematode growth and reproduction. Simultaneously, surrounding cortical cells undergo hypertrophy and hyperplasia, producing the characteristic root galls that serve as the major diagnostic symptom of RKN disease [2,8]. Successful establishment of this feeding site requires the nematode to manipulate host development while avoiding or suppressing multiple layers of plant immunity [9].

Plant immunity is generally organized into two interconnected branches: pattern-triggered immunity (PTI) and effector-triggered immunity (ETI) [10]. PTI is activated when cell surface-localized pattern recognition receptors (PRRs) perceive conserved microbe-associated molecular patterns (MAMPs), nematode-associated molecular patterns (NAMPs), or damage-associated molecular patterns (DAMPs). Ascarosides are a structurally diverse family of secreted nematode glycolipid signals rather than structural components of the nematode surface. In *Caenorhabditis elegans*, different ascarosides and ascaroside blends regulate dauer development, mating, aggregation, dispersal, and other developmental or social behaviors [11]. Ascr#18 is a long-chain ascaroside detected in the culture media of several plant-parasitic nematodes and can function as a nematode-associated molecular pattern in plants [12]. However, ascaroside profiles vary substantially among nematode species, developmental stages, population densities, and environmental conditions [13]. Ascr#18 should therefore be described as a secreted nematode chemical signal perceived by plants rather than as a structural molecule obligatorily exposed during J2 invasion. Whether an individual invading J2 releases sufficient ascr#18 to activate plant immunity at the infection site remains unknown. PTI activation triggers early defense events such as Ca^2+^ influx, mitogen-activated protein kinase (MAPK) cascade activation, transcriptional reprogramming, and rapid production of reactive oxygen species (ROS). This oxidative burst is mediated predominantly by plasma membrane-localized NADPH oxidases, also known as respiratory burst oxidase homologs (RBOHs), especially RBOHD [5,14]. ETI, in contrast, is activated when intracellular nucleotide-binding leucine-rich repeat receptors recognize specific pathogen or nematode effectors. Tomato Mi-1.2-mediated resistance provides a classic example of nucleotide-binding site (NBS)-leucine-rich repeat (LRR)-associated defense against RKNs [15].

ROS, particularly superoxide radicals (O_2_•^−^) and hydrogen peroxide (H_2_O_2_), occupy a central position at the interface between nematode perception and defense execution. H_2_O_2_ can act directly as an antinematodal molecule, and its toxicity toward migrating J2s has been reported in several plant–nematode systems [14,16]. Beyond direct toxicity, ROS function as diffusible signaling molecules that regulate salicylic acid (SA), jasmonic acid (JA), ethylene (ET), Ca^2+^-dependent signaling, lignin and callose deposition, systemic defense signaling, and hypersensitive response (HR)-associated programmed cell death (PCD) [6,14]. Thus, ROS are not merely by-products of stress metabolism but key regulators of immunity, development, and redox homeostasis during RKN infection.

However, the role of ROS in plant–RKN interactions is paradoxical and highly context dependent. In incompatible interactions, resistant plants often generate a sustained and amplified oxidative burst that promotes hypersensitive response-mediated cell death and restricts giant cell establishment, thereby arresting nematode development [6]. In compatible interactions, RKNs deploy secreted effectors that modulate host ROS production, antioxidant activity, and redox signaling to suppress defense-associated oxidative responses. At the same time, RBOH-derived ROS can also contribute to feeding site maintenance by preventing excessive cell death around developing giant cells, suggesting that ROS act as a “double agent” in nematode parasitism [17,18]. Accordingly, the outcome of plant–RKN interactions appears to depend not simply on the presence or absence of ROS, but on their concentration, subcellular origin, duration, spatial distribution, and interaction with antioxidant and hormonal networks.

This review synthesizes current molecular, biochemical, genetic, and transcriptomic evidence on ROS-mediated regulation of plant–RKN interactions. Specifically, it examines the machinery of ROS generation and scavenging in roots, the spatial and temporal dynamics of ROS during nematode-induced PTI, differential antioxidant enzyme regulation in resistant and susceptible hosts, effector-mediated manipulation of host redox homeostasis, hormone–ROS crosstalk, systemic ROS signaling, and the paradoxical pro-susceptibility role of RBOH-derived ROS. Finally, the review discusses how these insights may guide biotechnological strategies—including genetic engineering, genome editing, host-induced gene silencing, and chemical priming—for durable and environmentally sustainable nematode management.

## 2. Molecular Basis of ROS Homeostasis in Plant–Root-Knot Nematode Interactions

### 2.1. RBOH-Dependent ROS Generation and Upstream Immune Signaling

In plant roots, ROS are generated by multiple enzymatic and non-enzymatic systems. However, plasma membrane-localized NADPH oxidases, encoded by the *RBOH* gene family, constitute a major source of the rapid apoplastic oxidative burst induced by pathogen or nematode perception. Ten *AtRBOH* isoforms have been identified in *Arabidopsis thaliana*, among which RBOHD and RBOHF are the principal contributors to pathogen-triggered ROS production [5,19,20]. During immune activation, RBOHs initiate the apoplastic oxidative burst by transferring electrons from cytosolic NADPH to molecular oxygen at the outer surface of the plasma membrane. Because the primary product O_2_•^−^ and its downstream product H_2_O_2_ differ substantially in mobility, stability, reactivity, and biological function, distinguishing between these two species is essential for interpreting RBOH-dependent defense responses.

It is important to distinguish O_2_•^−^ from H_2_O_2_ rather than treating them as functionally interchangeable components of a homogeneous ROS pool. Plasma membrane-localized RBOHs transfer electrons from cytosolic NADPH to molecular oxygen on the apoplastic side of the membrane, thereby generating O_2_•^−^. This charged radical has very limited passive permeability across lipid bilayers and therefore generally acts close to its site of production. O_2_•^−^ can undergo spontaneous or superoxide dismutase (SOD)-catalyzed dismutation to H_2_O_2_, react rapidly with nitric oxide (NO), and participate in metal-dependent redox reactions. H_2_O_2_, by contrast, is a nonradical ROS that is more stable and longer-lived in biological systems. Its transport across biological membranes can be facilitated by aquaporins, including AtPIP2;1, enabling it to function as an intracellular and intercellular redox signal as well as an enzymatic substrate [21,22,23]. O_2_•^−^ and H_2_O_2_ should therefore not be considered functionally equivalent: O_2_•^−^ generally acts as a spatially restricted redox intermediate and local reactant, whereas H_2_O_2_ can function more readily as a mobile signal.

Plant roots illustrate the distinct but partially overlapping spatial distributions of these ROS. In *Arabidopsis*, O_2_•^−^ is relatively enriched in the meristematic or proliferative region, whereas H_2_O_2_ becomes more prominent toward the transition, elongation, and differentiation regions. The precise patterns vary with developmental stage, growth conditions, and detection method. This ROS gradient, which is regulated in part by UPBEAT1 and extracellular peroxidases (PODs), contributes to the balance between cell proliferation and differentiation [24,25]. The effects of these species on the cell wall are also context dependent. O_2_•^−^ can contribute to transition-metal redox cycling and indirectly support hydroxyl-radical (OH•) formation, whereas H_2_O_2_ is a direct substrate for Fenton reactions and class III PODs involved in extensin cross-linking and lignin polymerization. Consequently, cell-wall loosening or stiffening cannot be attributed solely to one ROS species but instead depends on local enzyme activity, transition-metal availability, ROS concentration, and cellular compartmentation.

These distinctions also have important methodological implications. Nitroblue tetrazolium (NBT) staining is widely used as a semiquantitative indicator of O_2_•^−^-dependent reduction and formazan formation, although it should not be considered absolutely specific without appropriate controls. CeCl_3_-based transmission electron microscopy localizes H_2_O_2_ through the formation of electron-dense cerium perhydroxide precipitates, whereas 3,3′-diaminobenzidine (DAB) staining reports POD-dependent H_2_O_2_-associated polymerization. 2′,7′-dichlorodihydrofluorescein diacetate (DCFH-DA)-based fluorescence is sensitive to oxidation by multiple reactive species and does not identify a single ROS or provide a specific quantitative measure of total ROS [6,26]. Signals obtained using NBT, CeCl_3_, DAB, and DCFH-DA should therefore not be interpreted interchangeably as equivalent measures of a unified ROS response.

This species-specific distinction is particularly relevant to plant–RKN interactions. The transient O_2_•^−^ pool generated at the apoplastic surface may influence local redox chemistry, NO-dependent reactions, and cell-wall remodeling during J2 migration and penetration. Conversion of O_2_•^−^ to H_2_O_2_ by plant or nematode SODs alters the spatial range and functional properties of the oxidative response rather than simply detoxifying O_2_•^−^. H_2_O_2_ can subsequently cross the plasma membrane, participate in SA-associated defense and HR signaling, or serve as a substrate for POD-mediated lignification. The dependence of callose deposition on RBOHD-derived ROS is elicitor- and context-dependent; callose accumulation should therefore not be attributed uniformly to H_2_O_2_ alone [27]. Organelle-derived ROS pools should also be distinguished. *Meloidogyne javanica* nematode effector ROS suppressor (Mj-NEROSs) alters plastid electron transport and the associated ROS output, whereas *Meloidogyne javanica* transthyretin-like protein 5 (MjTTL5) enhances H_2_O_2_ scavenging through the plastid ferredoxin–thioredoxin (FTR) system [7,28]. In the remainder of this review, H_2_O_2_ is specified when it was directly measured or specifically implicated, O_2_•^−^ is specified when supported by the analytical method or biochemical evidence, and the broader term “ROS” is retained only for nonspecific measurements or processes in which multiple reactive species may contribute. The respective contributions of O_2_•^−^ and H_2_O_2_ to toxicity toward invading nematodes, callose regulation, giant-cell development, and cell-wall remodeling remain important unresolved questions.

Against this biochemical and methodological background, RBOHD activation during immune perception is tightly regulated through coordinated Ca^2+^ binding and phosphorylation. Cytosolic Ca^2+^ binds to two EF-hand motifs in the N-terminal regulatory domain of RBOHD, directly enhancing its enzymatic activity. In parallel, perception by some cell-surface immune receptors activates BRASSINOSTEROID INSENSITIVE 1-ASSOCIATED RECEPTOR KINASE1 (BAK1)/SOMATIC EMBRYOGENESIS RECEPTOR KINASE 3 (SERK3)-containing receptor complexes and the receptor-like cytoplasmic kinase BOTRYTIS-INDUCED KINASE 1 (BIK1). Following ligand perception by BAK1-associated receptor complexes, BAK1 and the receptor complex regulate BIK1, which can directly phosphorylate RBOHD at Ser39 and Ser339 and thereby amplify PTI-associated ROS production [5,29]. The relevance of this signaling network to nematode immunity is supported by the enhanced susceptibility of *bik1* and *rbohD rbohF* mutant *Arabidopsis* plants to *M. incognita*, indicating that BIK1-dependent RBOH activation contributes to basal resistance against RKN infection [5].

BAK1/SERK3 is a shared co-receptor for multiple leucine-rich repeat receptor-like kinases, including immune receptors such as FLAGELLIN SENSING2 (FLS2) and EF-Tu RECEPTOR (EFR) and the brassinosteroid receptor BRI1 [30,31]. Because BAK1 participates in immune signaling, brassinosteroid-regulated growth, and cell-death control, phenotypes of BAK1-deficient plants must be interpreted cautiously in root-infection assays. Alterations in root growth, root architecture, or cell-death regulation may indirectly affect nematode penetration and feeding-site establishment independently of nematode-recognition signaling.

The *bak1-5* allele used by Teixeira et al. [5] contains a C408Y substitution in the BAK1 kinase domain and is best regarded as an immune-biased separation-of-function allele rather than as a null allele. It strongly compromises FLS2- and EFR-dependent immune signaling while largely preserving BRI1-dependent brassinosteroid (BR) signaling and overall development [32]. Thus, the enhanced susceptibility of *bak1-5* plants to *M. incognita* supports the conclusion that BAK1-dependent PTI contributes to basal RKN resistance. However, it does not demonstrate that BAK1 is strictly required for all nematode-triggered ROS production, nor does it identify the upstream receptor complex responsible for nematode perception.

The *bak1-4* single mutant is a viable BAK1 null allele. Severe growth defects, spontaneous cell death, or seedling lethality are associated primarily with higher-order SERK mutant combinations, such as *bak1 bkk1*, rather than with *bak1-4* alone [33,34]. Nevertheless, experiments using null or higher-order SERK mutants require controls carefully matched for root growth and architecture.

Beyond PRR-mediated signaling, RBOH activity is integrated with hormone-associated defense pathways. In tomato, brassinosteroid treatment enhances apoplastic ROS accumulation and activates MPK1/2 and MPK3 through an RBOH1-dependent pathway. Silencing *RBOH1*, *WFI1*, *MPK1*, *MPK2*, or *MPK3* increases susceptibility to *M. incognita*, demonstrating that the BR–RBOH1–MAPK cascade contributes positively to nematode resistance [35]. Consistent with isoform-specific regulation, resistant sweetpotato cultivars exhibit differential induction of *IbRboh* genes during *M. incognita* infection, suggesting that variation in the timing and magnitude of individual RBOH isoform responses may contribute to cultivar-level resistance [36,37].

The contribution of individual RBOH isoforms may also vary among plant–nematode pathosystems. In *Arabidopsis*, AtRBOHB has been implicated in resistance to the cyst nematode *Heterodera schachtii*, and its downregulation in developing syncytia suggests that sedentary nematodes may suppress selected RBOH isoforms to facilitate feeding-site establishment [16,38]. Together, these findings place RBOH-dependent ROS production at the convergence of pattern recognition, Ca^2+^ signaling, receptor-associated kinase activity, hormone signaling, and isoform-specific transcriptional regulation. However, the biological outcome of RBOH activation depends not only on the total magnitude of ROS production but also on the identity, localization, timing, and subsequent enzymatic processing of the ROS generated.

### 2.2. Secondary ROS Sources in the Cell Wall and Organelles

Although RBOHs are the dominant source of apoplastic ROS during immune activation, they do not act alone. Class III cell wall PODs can generate H_2_O_2_ through an NADH-dependent oxidative cycle and thereby contribute to both defense signaling and cell wall reinforcement [39]. In tomato–*M. incognita* interactions, pharmacological inhibition using diphenyleneiodonium (DPI), an NADPH oxidase inhibitor, showed that RBOH activity accounts for a major proportion of infection-induced ROS, whereas cyanide-sensitive PODs provide an additional ROS-generating component [6]. These two apoplastic ROS-producing systems likely operate cooperatively during the early stages of nematode penetration and feeding site initiation.

Subcellular organelles also contribute to ROS homeostasis. Chloroplasts, mitochondria, peroxisomes, and the endoplasmic reticulum produce ROS as by-products of electron transport, photorespiration, and redox reactions [40,41,42]. Peroxisomes are particularly important because they contain catalase, a highly efficient H_2_O_2_-degrading enzyme. Plastid-derived ROS have recently gained attention in plant–nematode interactions because the *M. javanica* effector Mj-NEROSs targets the plastid Rieske iron–sulfur protein, thereby manipulating chloroplastic electron transport and host redox balance [28,43]. These findings indicate that RKNs do not merely suppress plasma membrane-derived ROS but can also interfere with organelle-based redox networks.

### 2.3. Enzymatic Antioxidants Regulating ROS Homeostasis

The biological effect of ROS depends not only on their production but also on their scavenging. Plants maintain a complex antioxidant system that prevents uncontrolled oxidative damage while preserving ROS concentrations sufficient for defense signaling [40,41,42]. Superoxide dismutase (SOD) is the first major enzymatic component of this network, catalyzing the conversion of O_2_•^−^ to H_2_O_2_ and O_2_. Plant SODs are classified into Cu/Zn-SOD, Mn-SOD, and Fe-SOD isoforms, localized mainly in the cytosol, chloroplasts, and mitochondria. By converting O_2_•^−^ to H_2_O_2_, SOD reshapes the local redox environment; its net effect on defense depends on enzyme localization, timing, the functions of the O_2_•^−^ pool being depleted, and downstream H_2_O_2_-scavenging capacity [37,44,45].

Catalase (CAT) is a tetrameric heme-containing enzyme localized predominantly in peroxisomes, where it decomposes high concentrations of H_2_O_2_ into water and oxygen without requiring a reducing substrate. Because of its high catalytic efficiency, CAT functions as a major gatekeeper of cellular H_2_O_2_ levels. In resistant plant–RKN interactions, inhibition or reduced activity of CAT may allow H_2_O_2_ to accumulate to levels sufficient to activate HR-associated cell death and phenylpropanoid-mediated defense. Reduced CAT activity, together with pharmacological CAT inhibition by 3-amino-1,2,4-triazole, supports the view that limited H_2_O_2_-scavenging capacity can reinforce resistance. However, the extent to which physiological SA concentrations directly inhibit CAT in infected roots remains unresolved [14,18]. Infection-induced changes in CAT isozyme composition in tomato roots further suggest that RKN infection reshapes the qualitative as well as quantitative features of H_2_O_2_ detoxification.

Ascorbate peroxidase (APX) reduces H_2_O_2_ to water using ascorbate as a specific electron donor and operates in several cellular compartments, including the cytosol, chloroplast stroma, thylakoids, and peroxisomes [40,41,42]. In resistant sweetpotato cultivars, lower expression of cytosolic APX genes compared with susceptible cultivars is consistent with reduced H_2_O_2_ scavenging and greater defense-associated ROS accumulation [37]. PODs, commonly assayed using guaiacol or pyrogallol as substrates, play a dual role in plant defense by consuming H_2_O_2_ while oxidizing phenolic substrates, including monolignols, thereby linking ROS metabolism to lignin biosynthesis and cell wall fortification [39]. Elevated POD activity has been associated with resistance in sweetpotato, carrot, and tomato, suggesting that peroxidase-mediated lignification is a conserved structural defense against RKN invasion [8,45,46].

Glutathione peroxidases (GPXs) also participate in H_2_O_2_ and lipid hydroperoxide detoxification, using glutathione (GSH) as an electron donor [40,41,42]. In susceptible tomato roots, early upregulation of GPX genes during *M. incognita* infection may contribute to rapid H_2_O_2_ depletion and the establishment of a low-ROS environment favorable for nematode development [18]. Therefore, the relative activities of SOD, CAT, APX, POD, and GPX shape the cellular H_2_O_2_-associated redox state, which strongly influences whether ROS act as defense-promoting signals or are neutralized to support compatibility.

### 2.4. Non-Enzymatic Antioxidants and Redox Cycling Networks

In addition to enzymatic scavengers, non-enzymatic antioxidants provide essential buffering capacity. Ascorbate (AsA), GSH, carotenoids, tocopherols, and flavonoids modulate the effective concentration of ROS available for signaling, detoxification, and antimicrobial activity [14]. The AsA–GSH cycle is central to this process. In this cycle, APX uses ascorbate to reduce H_2_O_2_, dehydroascorbate reductase (DHAR) regenerates AsA using reduced GSH, and glutathione reductase (GR) converts oxidized GSH back to its reduced form using NADPH. Thus, redox cycling is closely connected to cellular NADPH availability.

GSH-associated enzymes are also involved in nematode defense. Glutathione S-transferases (GSTs) are differentially regulated during RKN infection, and several GST-encoding genes are upregulated in resistant tomato roots infected with *M. incognita*, indicating a role in oxidative stress tolerance and detoxification [8]. The GSH/thioredoxin (TRX) system, including the FTR complex, contributes to plastid redox homeostasis and is exploited by the RKN effector MjTTL5 to enhance H_2_O_2_ scavenging and suppress plant defense [7]. Furthermore, cytosolic glucose-6-phosphate dehydrogenase (G6PDH), which generates NADPH through the oxidative pentose phosphate pathway, supports RBOH-driven ROS production. *Arabidopsis g6pd5/6* mutants exhibit reduced ROS accumulation, impaired defense gene expression, and increased susceptibility to RKN infection, demonstrating that NADPH supply is an integral component of ROS-mediated basal defense [16].

**Figure 1:**
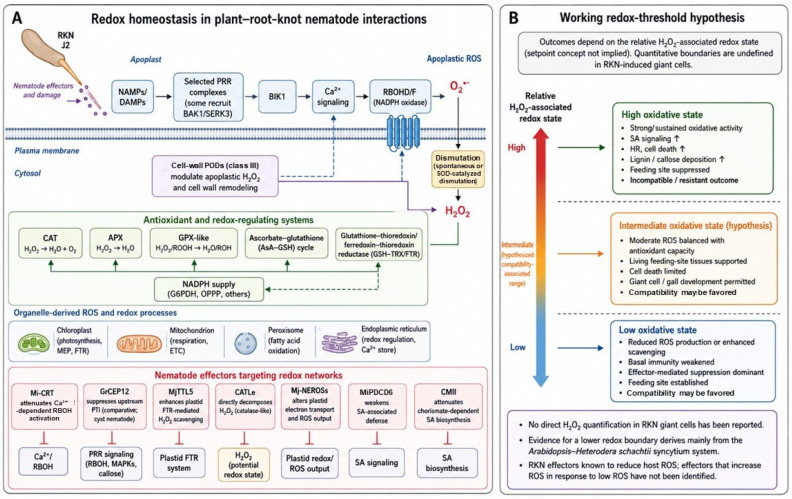
Integrated framework for ROS homeostasis in plant–root-knot nematode interactions. (**A**) Root-knot nematode (RKN) invasion causes mechanical damage and releases nematode- or damage-associated molecular cues that are perceived by selected cell-surface receptor complexes, some of which recruit BAK1/SERK3. Downstream BIK1 and Ca^2+^ signaling activate RBOHD/F, which directly generates apoplastic O_2_•^−^. O_2_•^−^ is subsequently converted to H_2_O_2_ through spontaneous or superoxide dismutase (SOD)-catalyzed dismutation. H_2_O_2_ dynamics are further shaped by class III cell-wall peroxidases, organelle-derived ROS, catalase (CAT), ascorbate peroxidase (APX), glutathione peroxidase (GPX), the ascorbate–glutathione cycle, the glutathione–thioredoxin/ferredoxin–thioredoxin reductase system, and cellular NADPH supply. RKN effectors act at multiple levels: Mi-CRT attenuates Ca^2+^-dependent RBOH activation, MjTTL5 enhances plastid FTR-linked H_2_O_2_ scavenging, CATLe directly decomposes H_2_O_2_, Mj-NEROSs alters plastid electron transport and ROS output, and MiPDCD6 and CMII attenuate SA-associated defense. GrCEP12 is included only as comparative evidence from a cyst nematode. (**B**) Working redox-threshold hypothesis. Strong or sustained oxidative activity is associated with defense and frequently with incompatible outcomes, whereas reduced ROS production or enhanced scavenging can weaken basal immunity and favor compatibility. An intermediate compatibility-associated redox state is hypothesized to support living feeding-site tissues while limiting excessive cell death; however, its quantitative boundaries and any lower H_2_O_2_ requirement have not been demonstrated in RKN-induced giant cells. Evidence for a lower redox boundary derives mainly from the *Arabidopsis–Heterodera schachtii* syncytium system. The states shown are therefore relative and do not represent defined H_2_O_2_ concentrations or a nematode-controlled fixed setpoint.

Collectively, ROS homeostasis in plant–RKN interactions is shaped by the dynamic balance among ROS-generating systems, antioxidant enzymes, non-enzymatic redox buffers, organelle-specific redox processes, and nematode effectors that manipulate these pathways (Figure 1). Resistance reflects the spatially and temporally coordinated production, conversion, transport, and scavenging of distinct ROS rather than simply high ROS abundance. Compatible interactions often involve attenuation or redistribution of defense-associated ROS together with enhanced antioxidant buffering. The intermediate, compatibility-associated redox state illustrated in Figure 1B should be regarded as a working hypothesis, because its quantitative H_2_O_2_ boundaries—and the existence of a lower H_2_O_2_ requirement in RKN-induced giant cells—have not yet been established.

## 3. PTI-Mediated ROS Dynamics Determine Compatibility in Plant–Root-Knot Nematode Interactions

### 3.1. Early Recognition of RKNs and the Initial Oxidative Burst

RKN invasion rapidly activates PTI responses in plant roots, including the production of ROS. The earliest oxidative burst appears to be a common response in both compatible and incompatible interactions, reflecting mechanical damage caused by J2s and/or the perception of NAMPs. In tomato roots infected with *M. incognita*, ROS accumulation was detected within the first 12 h after inoculation by fluorometric DCFH-DA assays and histochemical approaches using NBT and CeCl_3_ [6]. CeCl_3_-based transmission electron microscopy further showed that H_2_O_2_ accumulated mainly at the plasma membrane and cell wall near the interface between invading J2s and host root cells, indicating that ROS production is spatially focused at the site of nematode penetration. The strong inhibition of H_2_O_2_ accumulation by DPI, an NADPH oxidase inhibitor, confirmed that plasma membrane-localized RBOH enzymes are the major source of the early ROS burst [6,47].

Genetic analyses in *Arabidopsis thaliana* indicate that BAK1-dependent receptor signaling, BIK1, and RBOHD/RBOHF contribute to basal resistance against *M. incognita* [5]. In particular, the enhanced susceptibility of *bak1-5* plants supports a role for BAK1-dependent PTI in nematode defense. However, because *bak1-5* is an immune-biased separation-of-function allele and BAK1 is shared by multiple receptor complexes, this phenotype neither identifies the upstream nematode-responsive receptor nor demonstrates that BAK1 is strictly required for all nematode-triggered ROS production. The enhanced susceptibility of *bik1* and *rbohD rbohF* mutants nevertheless supports important roles for receptor-like cytoplasmic kinase signaling and RBOH-dependent ROS production in basal RKN resistance. Pretreatment with flg22 also reduces J2 invasion, indicating that prior activation of PTI can enhance resistance; however, this result does not identify FLS2 as a receptor for an RKN-derived ligand. PTI-associated genes, including *CYP71A12*, *MYB51*, *WRKY11*, and *WRKY17*, are also implicated in early nematode defense responses.

However, NILR1-dependent ROS production elicited by crude nematode aqueous extracts should not be attributed exclusively to ascr#18. Mendy et al. [48] concluded that the eliciting activity in these extracts was proteinaceous, whereas direct binding of purified ascr#18 to the NILR1 ectodomain was demonstrated subsequently by Huang et al. [49]. Moreover, purified ascr#18 does not consistently induce a canonical rapid ROS burst in *Arabidopsis* roots [50]. Responses to crude nematode extracts and purified ascr#18 should therefore be distinguished, and ascr#18-mediated resistance should not be equated uniformly with RBOH-dependent ROS production, as discussed further in Section 3.3.

### 3.2. Temporal Divergence Between Compatible and Incompatible Interactions

Although both resistant and susceptible hosts initiate an early ROS burst, the decisive difference lies in the subsequent temporal trajectory of ROS accumulation. Between 12 and 48 h post-inoculation, incompatible interactions develop a sustained second phase of ROS production, whereas compatible interactions show a marked attenuation of ROS levels [6]. In resistant tomato plants challenged with avirulent *M. incognita*, H_2_O_2_ accumulation remains elevated or increases further during this period, coinciding with HR-associated cell death, arrest of nematode migration, callose deposition, and activation of phenylpropanoid-based defenses. This biphasic pattern resembles the two-phase oxidative burst commonly observed in incompatible plant–pathogen interactions.

In contrast, compatible interactions are characterized by a rapid decline in H_2_O_2_ after the initial burst. By 48–72 h post-inoculation, ROS levels in susceptible roots may fall below those of uninfected controls, generating a low-ROS environment that permits giant cell induction and feeding site establishment [6,18]. Subcellular localization studies support this distinction. In incompatible interactions, H_2_O_2_ deposits progress from the plasma membrane, cell wall, and intercellular spaces into the cytoplasm and vacuoles of cells undergoing hypersensitive cell death. In compatible interactions, CeCl_3_ deposits are weaker and gradually disappear from the plasma membrane as giant cells develop. These observations indicate that successful nematode parasitism depends not only on suppressing ROS production but also on reshaping the spatial distribution of H_2_O_2_ around developing feeding sites.

### 3.3. Molecular Recognition of Nematode- and Damage-Associated Signals

#### 3.3.1. Ascaroside Perception, Concentration Dependence, and Cross-Kingdom Chemical Signaling

Among nematode ascarosides, ascr#18 is the best-characterized plant-active signal. Its classification as a NAMP does not imply that it is a structural surface component analogous to flagellin or chitin. Rather, it is a secreted long-chain ascaroside pheromone or exometabolite produced by several nematode species. Plants can exploit such chemical signals as indicators of nematode presence, potentially before or during root invasion [11,12].

Direct molecular evidence supports perception of ascr#18 by NILR1. Mendy et al. [48] initially showed that NILR1 was required for immune responses to nematode-derived aqueous extracts, although the eliciting activity in those extracts was characterized as proteinaceous. Huang et al. [49] subsequently demonstrated direct binding between ascr#18 and the NILR1 ectodomain and showed that *nilr1* mutants were impaired in ascr#18-induced signaling and resistance. Whether *Arabidopsis* NILR1 recruits BAK1/SERK3 or another co-receptor during ascr#18 signaling remains unresolved. In potato, StNILR1 recognizes both ascr#18 and brassinosteroid and signals through StBAK1; the *Globodera pallida* effector RHA1B targets StNILR1 for ubiquitination and proteasome-dependent degradation [50]. This potato–cyst-nematode system provides comparative mechanistic evidence but does not establish the co-receptor requirement of *Arabidopsis* NILR1 or constitute direct evidence from an RKN interaction. The physiological relevance of ascr#18 during natural J2 invasion remains quantitatively unresolved. Manosalva et al. [12] detected approximately 5–100 nM ascr#18 in culture media from *Meloidogyne* species and reported biological activity of synthetic ascr#18 over a picomolar-to-micromolar range, depending on the plant species and experimental endpoint. However, culture-medium measurements integrate release from many nematodes over time. Neither the amount released by an individual J2 nor the local concentration reached at the root surface or within invaded tissue has been measured directly. Consequently, evidence that externally applied ascr#18 activates plant responses does not establish that endogenous ascr#18 reaches the same concentration or spatial distribution during natural infection.

The downstream responses to ascr#18 also depend on tissue, treatment regime, concentration, host species, and measured endpoint. Although NILR1-dependent immune signaling has been demonstrated, purified ascr#18 does not invariably induce a canonical rapid ROS burst. Letia et al. [51] reported that ascr#18 treatment of *Arabidopsis* roots did not induce typical ROS-burst or defense-related growth-inhibition responses. Instead, ascr#18 reduced auxin transport and signaling, including the expression of *AUX1*, *SAUR69*, and *IAA27*, thereby interfering with processes required for cyst-nematode feeding-site development. These auxin-related responses occurred independently of NILR1. Ascr#18-mediated resistance may therefore involve multiple outputs, including NILR1-dependent immune perception and NILR1-independent suppression of auxin-associated susceptibility, rather than a single linear NILR1–RBOH–ROS pathway. Whether this auxin-related mechanism also operates during RKN-induced giant-cell development remains unknown.

Recent evidence further expands the known outputs of ascr#18 perception. Manohar et al. [52] showed that exposure of seeds or plants to ascr#18 primes defense genes for enhanced activation following subsequent microbial pathogen challenge and that this priming is associated with increased chromatin accessibility at defense-gene regulatory regions. Priming and disease protection were compromised in *nilr1* mutants, supporting a NILR1-dependent priming mechanism. However, this study did not examine natural RKN invasion, did not determine the local ascr#18 concentration released by individual invading J2s, and did not establish ROS as the principal downstream signal. The priming response should therefore be regarded as an additional context-dependent output of ascr#18 perception rather than as evidence for a universal NILR1–RBOH–ROS pathway during RKN infection [52].

Ascaroside-mediated interactions extend beyond direct NAMP perception. Plants metabolize ascr#18 through peroxisomal β-oxidation to produce shorter-side-chain metabolites, including ascr#10 and ascr#9 [53]. Ascr#9, alone or in a mixture with ascr#18, repels *Meloidogyne* J2s in behavioral assays, and plant conversion of ascr#18 into shorter-chain metabolites is associated with reduced nematode infection. Candidate *M. incognita* genes involved in the behavioral response to ascr#9 have been identified, although the primary sensory receptor remains unknown [54]. This pathway represents metabolic editing of nematode chemical communication rather than classical immune-receptor signaling. In contrast, direct evidence that ascr#10 activates plant immunity or independently alters RKN infection remains limited. Determining which ascarosides are released by infective J2s, their concentrations at the root interface, their cognate plant and nematode receptors, and their ROS-dependent and ROS-independent outputs remains an important research priority.

#### 3.3.2. Potential BAK1-Independent and Damage-Responsive Inputs into RBOH Activation

The LYSIN MOTIF RECEPTOR-LIKE KINASE5 (LYK5)–CHITIN ELICITOR RECEPTOR KINASE1 (CERK1) complex provides a potential BAK1-independent route for pattern-triggered ROS production. In *Arabidopsis*, LYK5 and CERK1 mediate chitin perception without requiring BAK1 and activate immune signaling that can converge on receptor-like cytoplasmic kinases and RBOHD-dependent ROS production [29,55]. However, no RKN-derived ligand has been shown to activate LYK5–CERK1 during root invasion. The presence of chitin in nematode eggshells does not demonstrate that a CERK1-activating ligand is exposed or released during migration of infective J2s through root tissue. Direct CERK1-dependent ROS production in RKN-infected roots also remains unproven. The LYK5–CERK1 pathway should therefore be regarded as a candidate parallel route rather than as an established compensatory mechanism for reduced BAK1 signaling.

Damage-associated signaling may provide an additional input into RBOHD regulation. P2-purinoceptor Kinase1 (P2K1)/DOes not Respond to Nucleotides1 (DORN1) is a lectin receptor kinase that perceives extracellular ATP and mediates ATP-induced Ca^2+^ signaling. In other plant contexts, P2K1/DORN1 directly phosphorylates RBOHD and promotes ROS production [56,57]. Although the *dorn1* mutant examined by Teixeira et al. [5] did not exhibit enhanced susceptibility under the conditions tested, mechanical disturbance caused by migrating J2s could release ATP into the root apoplast. Whether P2K1/DORN1 contributes redundantly to damage-triggered ROS production during RKN penetration therefore remains unknown.

GLR family members should be interpreted in a species- and context-specific manner. In *Arabidopsis thaliana*, GLR3.3 and GLR3.6 mediate wound-induced long-distance Ca^2+^ signaling [58], but these channels have not been functionally tested during RKN infection and have not been shown to activate RBOHD in nematode-infected roots. They should therefore be considered candidate damage-responsive signaling components rather than established elements of local RKN-triggered PTI. By contrast, genetic and grafting evidence in tomato supports a role for GLR3.5 in RKN-induced systemic root-to-shoot electrical and ROS signaling and JA-associated resistance, as discussed in Section 7.5 [59]. The *Arabidopsis* GLR3.3/GLR3.6 wound-signaling pathway and the tomato GLR3.5 RKN systemic-signaling pathway should therefore not be conflated.

Current evidence is therefore consistent with a multi-input model in which BAK1-dependent receptor signaling contributes to basal RKN resistance, whereas LYK5–CERK1-mediated pattern recognition and P2K1/DORN1- or GLR3.3/GLR3.6-mediated damage signaling may provide candidate parallel inputs into Ca^2+^ signaling, receptor-like cytoplasmic kinase activity, and RBOH regulation. These candidate local pathways should be distinguished from the GLR3.5–RBOH1-dependent systemic signaling pathway demonstrated in tomato. Whether the proposed local pathways function redundantly or partially compensate for reduced BAK1 signaling during RKN infection remains unresolved.

### 3.4. Suppression of SA-Dependent ROS Defense in Compatible Interactions

In susceptible hosts, RKNs actively suppress SA-dependent defense pathways that normally reinforce ROS accumulation. SA signaling can be associated with reduced H_2_O_2_-scavenging capacity and altered CAT or APX activity, thereby favoring H_2_O_2_ accumulation under some conditions; however, direct enzyme inhibition is context-dependent [14]. During early *M. incognita* infection in susceptible tomato, SA-responsive defense genes, including pathogenesis-related (*PR*)*-1*, *PR-2*, *PR-4b*, and *PR-5*, are significantly downregulated as early as 3 days post-inoculation. This indicates that RKNs suppress the SA branch of immunity at the onset of feeding site establishment.

Pharmacological evidence supports the importance of SA-mediated redox defense. Reducing SA levels with paclobutrazol increases nematode infection, whereas treatment with methyl salicylate decreases galling and nematode reproduction by approximately 50% [18]. One potential mechanism for SA suppression involves the *M. incognita* chorismate mutase effector CMII, which can divert chorismate away from SA biosynthesis toward prephenate production, thereby weakening SA-dependent immune signaling [8,18]. Notably, JA- and ET-related genes such as *JERF3* and *ACO* are not strongly affected during early infection, suggesting that RKN-mediated immune suppression is preferentially directed toward the SA-associated ROS defense pathway.

### 3.5. Antioxidant Activation as a Compatibility Strategy

The attenuation of ROS in compatible interactions is closely linked to the activation of host antioxidant systems. In susceptible tomato roots infected with *M. incognita*, transcripts encoding CAT and GPX are significantly induced, while enzymatic activities of SOD, CAT, and APX increase during the early stages of infection, particularly between 3 and 7 days post-inoculation [18]. Isoelectro focusing (IEF) analysis has also revealed the appearance of novel neutral SOD isoforms in infected tomato roots, suggesting that RKN infection recruits specific antioxidant isoenzyme profiles to regulate local ROS concentrations.

Chemical manipulation of ROS levels provides causal evidence for the role of redox balance in determining disease outcome. ROS-lowering treatments such as diphenyleneiodonium, which inhibits NADPH oxidase, and N,N′-dimethylthiourea (DMTU), a chemical ROS scavenger, increase galling and nematode reproduction. Conversely, ROS-elevating treatments such as 3-amino-1,2,4-triazole, a CAT inhibitor, and diethyldithiocarbamic acid (DIECA), which perturbs superoxide detoxification, significantly reduce infection severity [18]. These findings demonstrate that elevated ROS, particularly H_2_O_2_, can shift susceptible interactions toward a more resistant state, whereas excessive antioxidant activity favors compatibility by keeping ROS below the threshold required for effective defense.

**Figure 2:**
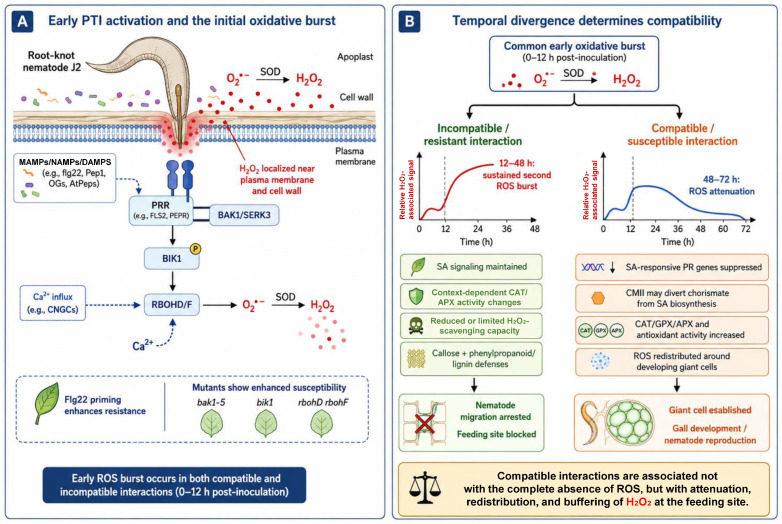
PTI-mediated ROS dynamics determine compatibility in plant–root-knot nematode interactions. (**A**) Root-knot nematode (RKN) invasion triggers early pattern-triggered immunity (PTI), in which NAMP/DAMP perception activates selected cell-surface receptor pathways, including some BAK1/SERK3-associated complexes, which can converge on BIK1, Ca^2+^ signaling, and RBOHD/F. RBOHD/F generates apoplastic O_2_•^−^, which is converted to H_2_O_2_ by superoxide dismutase (SOD). This early oxidative burst occurs in both compatible and incompatible interactions and is localized mainly near the plasma membrane and cell wall at the nematode penetration site. (**B**) After the shared early ROS burst, resistant and susceptible interactions diverge. In incompatible interactions, sustained H_2_O_2_ accumulation between 12 and 48 h post-inoculation is associated with SA signaling, reduced antioxidant capacity, and CAT/APX activity changes, hypersensitive response-associated cell death, and callose/lignin-mediated defenses, thereby blocking feeding site establishment. In compatible interactions, H_2_O_2_ is attenuated and redistributed through suppression of SA-responsive genes and activation of antioxidant systems, allowing giant cell formation, gall development, and nematode reproduction.

Collectively, ROS dynamics during plant–RKN interactions are governed by a transition from a shared early PTI-associated oxidative burst to divergent redox programs (Figure 2). Resistant interactions maintain or amplify ROS accumulation, leading to hypersensitive cell death and structural defense reinforcement. Susceptible interactions suppress SA-dependent defense and activate antioxidant pathways, thereby reducing H_2_O_2_ and permitting giant cell development. Therefore, compatibility is not determined by the absence of ROS, but by the nematode’s ability to attenuate, redistribute, or buffer ROS at the feeding site.

## 4. Redox Enzyme Reprogramming as a Determinant of Resistance and Susceptibility in Plant–RKN Interactions

### 4.1. The SOD–CAT Balance as a Context-Dependent Redox Determinant

In several plant–RKN systems, resistant and susceptible interactions are associated with contrasting regulation of the SOD–CAT/APX balance, although this pattern is not universal across hosts, nematode species, tissues, or infection stages. In several reported resistant pathosystems, elevated SOD activity combined with limited or unchanged CAT/APX activity is associated with greater H_2_O_2_ accumulation; however, this pattern is not universal. Comparative studies in sweet potato, tomato, and carrot indicate that this SOD–CAT balance is a major determinant of whether ROS function as resistance-promoting signals or are neutralized to favor compatibility.

The net effect of SOD induction on plant defense is highly context-dependent because SOD simultaneously removes O_2_•^−^ and generates H_2_O_2_. In resistant interactions in which H_2_O_2_-scavenging enzymes such as CAT and APX are suppressed or are not concomitantly induced, elevated SOD activity can shift the local redox balance toward H_2_O_2_ accumulation and may reinforce H_2_O_2_-dependent defense signaling, phenylpropanoid metabolism, and lignification [14,45]. By contrast, in susceptible interactions in which SOD is co-induced with CAT and/or APX, SOD-derived H_2_O_2_ may be rapidly removed, thereby contributing to redox buffering rather than necessarily enhancing defense. Moreover, SOD-mediated depletion of O_2_•^−^ may attenuate the independent local functions of O_2_•^−^ in redox signaling, cell-death regulation, localized cytotoxic reactions, and interactions with NO [60,61]. However, the contributions of these O_2_•^−^-dependent processes to RKN resistance remain poorly resolved and should not be inferred solely from measurements of SOD activity.

Nematode-derived and effector-mediated modulation of SOD activity further illustrates this context dependence. Virulent *M. incognita* J2s exhibit higher MnSOD expression and activity than their avirulent counterparts, suggesting that nematode SOD contributes to oxidative-stress tolerance and virulence [62]. Similarly, the cyst nematode *G. pallida* expresses a putatively secreted Cu/Zn-SOD, SOD-3, during early parasitism, with expression patterns that vary among susceptible, resistant, and immune hosts [63]. Although this finding derives from a cyst-nematode system rather than an RKN system, it provides comparative evidence that sedentary endoparasitic nematodes can deploy their own SOD systems during infection. In rice, the *M. graminicola* effector MgMO289 interacts with OsHPP04, increases host cytosolic Cu/Zn-SOD2 activity, lowers O_2_•^−^ levels, and suppresses immunity [64]. These findings demonstrate that increased SOD activity can be associated with either host resistance or nematode parasitism. High SOD activity should therefore not be regarded as an intrinsic marker of resistance; its biological outcome depends on the origin and subcellular localization of the enzyme, the timing of its induction, the downstream fate of SOD-generated H_2_O_2_, and the biological functions of the O_2_•^−^ pool being depleted.

In sweetpotato (*Ipomoea batatas*), resistant cultivars such as Danjami, Pungwonmi, and Juhwangmi exhibit higher basal SOD activity and H_2_O_2_ levels than susceptible cultivars such as Dahomi, Shinhwangmi, and Yulmi. Following *M. incognita* infection, resistant cultivars rapidly accumulate H_2_O_2_ within 7 days post-infection, whereas susceptible cultivars show a delayed H_2_O_2_ increase only at later stages, such as 28 days post-infection [45]. This early H_2_O_2_ accumulation in resistant cultivars is associated with elevated expression of phenylpropanoid pathway genes, including *PAL1*, *PAL4*, and *CAD*, as well as increased total phenolic and lignin contents. By contrast, susceptible cultivars induce both SOD and CAT, effectively buffering H_2_O_2_ and preventing timely activation of defense responses.

In tomato, the *Mi-1.2*-mediated incompatible response provides evidence for a resistance-associated reduction in CAT activity. Molinari and Leonetti [14] reported that CAT activity was transiently reduced in resistant tomato roots between 1 and 5 days after inoculation with avirulent *M. incognita*, whereas CAT activity remained unchanged or increased during compatible interactions. Isolated CAT-containing fractions were partially inhibited in vitro by relatively high concentrations of SA or H_2_O_2_; however, these assays do not establish direct SA binding as the predominant cause of CAT suppression in infected roots. The observed reduction in CAT activity is therefore best interpreted as part of a broader, context-dependent decrease in H_2_O_2_-scavenging capacity, as discussed in Section 4.3. Methyl salicylate treatment reduces galling and nematode reproduction, whereas pharmacological reduction in SA enhances infection, supporting a role for SA signaling in resistance without demonstrating direct SA-mediated CAT inhibition [18].

A similar pattern has been reported in carrot (*Daucus carota*) during *M. javanica* infection. In the resistant cultivar TN-99-129, CAT activity decreases early after infection, while SOD and POD activities increase at later stages. In the susceptible cultivar TN-99-62, CAT activity increases instead, limiting H_2_O_2_ accumulation [46]. The resistant carrot response follows a sequential model: early CAT inhibition permits H_2_O_2_ accumulation, H_2_O_2_ promotes cell death and defense signaling, SOD sustains ROS conversion toward H_2_O_2_, and POD activity supports callose and lignin deposition. APX activity remains largely unchanged in both resistant and susceptible carrot cultivars, suggesting that APX is not always the principal discriminator of resistance outcome in this system.

### 4.2. POD as a Bridge Between ROS Detoxification and Cell Wall Reinforcement

POD, often measured as activity depending on assay context, plays a dual role in plant–RKN interactions. On one hand, it removes H_2_O_2_ and prevents uncontrolled oxidative injury. On the other hand, it uses H_2_O_2_ to oxidize phenolic substrates, including monolignols such as coniferyl, sinapyl, and p-coumaryl alcohols, thereby promoting lignin polymerization in the cell wall. Thus, POD links ROS metabolism directly to structural defense against nematode migration, stylet penetration, and feeding site establishment.

In resistant sweetpotato cultivars, POD activity is consistently higher than in susceptible cultivars throughout infection and correlates with elevated phenolic and lignin accumulation [45]. This suggests that resistant plants do not simply accumulate ROS indiscriminately; instead, they channel H_2_O_2_ into phenylpropanoid metabolism and cell wall fortification. Transcriptomic evidence further supports this interpretation, as sweetpotato resistant cultivars show higher expression of redox-related genes, including SOD isoforms, glutaredoxin-related genes, cytochrome P450 genes, and defense-associated PODs [37].

In carrot, increased POD activity from 6 days post-infection coincides with callose deposition and precedes later lignin accumulation at 21 days post-infection [45]. This temporal sequence suggests that POD-mediated H_2_O_2_ utilization coordinates early ROS buffering with later physical reinforcement. Because H_2_O_2_ can also regulate callose synthase activity and plasmodesmatal permeability, POD may contribute not only to lignification but also to symplastic restriction around developing feeding sites.

### 4.3. Context-Dependent Regulation of H_2_O_2_ Scavenging by SA

SA is an important regulator of the ROS–antioxidant balance during plant immunity, but its effects on H_2_O_2_-scavenging enzymes should not be reduced to a single mechanism of direct CAT inhibition. Early biochemical studies identified tobacco catalase as an SA-binding protein and showed that SA could inhibit CAT activity in vitro, leading to the proposal that SA promotes H_2_O_2_ accumulation by directly restricting H_2_O_2_ degradation [65]. However, subsequent evidence indicates that the physiological relevance of this mechanism is strongly context-dependent. In tobacco extracts, measurable CAT inhibition required relatively high SA concentrations, and SA-induced *PR-1a* expression occurred in leaf tissue without detectable inhibition of CAT activity [66]. In *Arabidopsis*, early SA-induced H_2_O_2_ accumulation was associated with enhanced Cu/Zn-SOD activity and preceded substantial inhibition of CAT or APX, whereas later decreases in antioxidant-enzyme activity were associated with prolonged SA exposure and oxidative injury [67]. Elevated SA can also alter H_2_O_2_ and glutathione homeostasis without a corresponding reduction in CAT activity [68]. Direct SA-mediated CAT inhibition should therefore be regarded as a possible context-specific contribution rather than as a universal initiating mechanism of the SA–H_2_O_2_ relationship.

APX may represent a more sensitive biochemical target of SA under some conditions. Cytosolic APX was inhibited by SA with an IC_50_ of approximately 78 µM under the assay conditions used by Durner and Klessig [69], whereas pronounced CAT inhibition generally required higher SA concentrations. Nevertheless, this difference in vitro sensitivity does not establish APX as the principal SA target in vivo. SA-associated H_2_O_2_ accumulation may instead reflect varying combinations of direct enzyme inhibition, altered abundance or turnover of antioxidant enzymes, changes in reductant availability, and broader redox-dependent immune signaling [70,71].

In plant–RKN interactions, resistant tomato and sweetpotato cultivars exhibit reduced H_2_O_2_-scavenging capacity and greater H_2_O_2_ accumulation than susceptible cultivars during incompatible interactions [14,45]. These findings are consistent with a defense-associated shift in antioxidant capacity but do not establish direct inhibition of CAT or APX by physiological SA concentrations in infected roots. Although isolated CAT-containing fractions from tomato roots were partially inhibited in vitro by relatively high concentrations of SA or H_2_O_2_, the mechanism responsible for CAT suppression in infected tissue remains unresolved [14]. Accordingly, the SA–H_2_O_2_ relationship in resistant plant–RKN interactions is best interpreted as a network-level regulatory association in which SA-dependent immune signaling and reduced H_2_O_2_-scavenging capacity may reinforce one another, rather than as a feedback loop mediated exclusively by direct SA binding to CAT.

### 4.4. Isoform-Specific and Transcriptome-Level Regulation of Antioxidant Enzymes

Antioxidant regulation during RKN infection is not limited to total enzyme activity; it also involves isoform-specific changes with distinct subcellular localization and kinetic properties. In tomato, isoelectrofocusing has revealed novel neutral SOD isoforms in susceptible roots during early *M. incognita* infection, suggesting that specific SOD isoenzymes may be recruited to regulate ROS at the plant–nematode interface [18]. Multiple CAT isoforms in tomato also differ in their sensitivity to SA and H_2_O_2_, indicating that CAT regulation is both quantitative and qualitative [14].

Transcriptomic analyses provide broader support for this isoform-level model. In sweetpotato, resistant cultivars express genes encoding Cu/Zn-SOD, Fe-SOD, and Mn-SOD at higher levels than susceptible cultivars under both control and infected conditions. In contrast, genes encoding CAT and cytosolic APX tend to be expressed at lower levels in resistant cultivars, consistent with reduced H_2_O_2_ scavenging and a primed pro-defense redox state [37]. These findings suggest that resistance can be partially preconfigured before infection through constitutive differences in the ROS-regulatory transcriptome.

Tomato transcriptome analysis also reveals complex stage-specific regulation of redox genes. In resistant *Mi-1.2*-containing tomato, receptor-like kinase (RLK) genes, protein phosphatase 2C (PP2C), and SA-, ABA-, and ET-related defense components are activated during early infection stages, consistent with early immune signaling before or during oxidative burst development [8]. Interestingly, nematode-derived antioxidant-related genes, including CAT, GST, and C-type lectin genes, are also induced during resistant interactions, suggesting that nematodes mount their own antioxidant response to survive elevated host ROS.

### 4.5. Host-Species Variation and Exceptions to the ROS–HR Model

Although the SOD–CAT–H_2_O_2_ model is broadly conserved, resistance mechanisms vary among host species. In *Arabidopsis thaliana*, mutant analyses show that *bak1-5*, *bik1*, and *rbohD rbohF* plants are more susceptible to *M. incognita*, demonstrating that PTI-associated ROS production is required for basal RKN resistance [5]. Defense-related genes such as *CYP71A12*, *MYB51*, *WRKY11*, and *WRKY17* are also associated with nematode-induced defense responses, further linking immune signaling to ROS-mediated resistance.

Cowpea (*Vigna unguiculata*) provides an important exception to the classical ROS-driven HR model. In near-isogenic resistant CB46 plants carrying the *Rk* gene and susceptible null-*Rk* plants, both genotypes show similar J2 penetration rates and comparable early NADPH oxidase-dependent oxidative bursts during the first 24–48 h after infection [47]. However, resistant cowpea does not display a typical biphasic ROS burst or visible classical HR during early infection. Instead, giant cells initially form but later undergo vacuolation, cytoplasmic deterioration, and collapse by approximately 21 days post-infection. This indicates that *Rk*-mediated resistance functions as a delayed post-feeding-site resistance mechanism rather than an immediate ROS-HR. Therefore, ROS magnitude alone does not determine resistance; timing, localization, cellular context, and downstream developmental consequences are equally critical.

**Figure 3:**
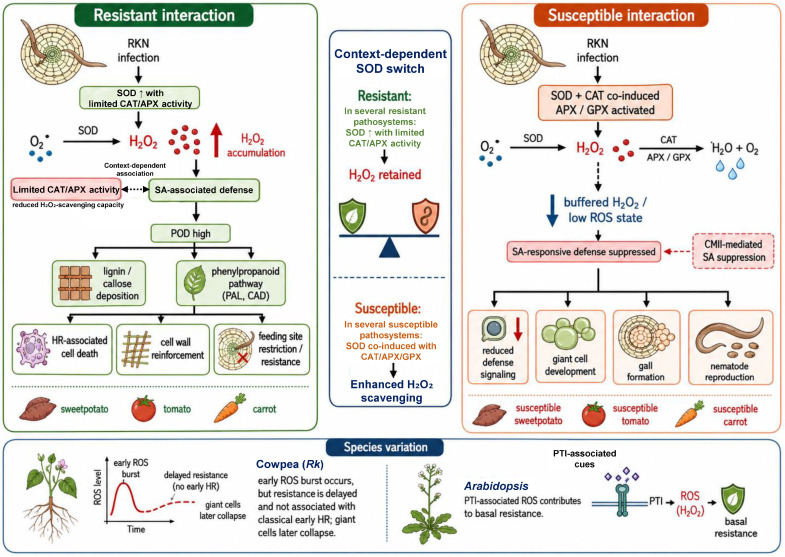
Redox enzyme reprogramming determines resistance and susceptibility in plant–RKN interactions. In several reported plant–RKN systems, resistance is associated with elevated SOD activity together with limited or unchanged H_2_O_2_-scavenging capacity, whereas susceptible interactions may co-induce SOD and downstream scavenging enzymes. Sustained H_2_O_2_ is associated with SA signaling and reduced H_2_O_2_-scavenging capacity, and activates POD-mediated lignin/callose deposition and phenylpropanoid defenses, leading to HR-associated cell death, cell wall reinforcement, and feeding site restriction. In susceptible interactions, coordinated induction of SOD, CAT, APX, and GPX buffers H_2_O_2_ and maintains a low-ROS state. This redox environment, together with suppression of SA-responsive defense, promotes giant cell development, gall formation, and nematode reproduction. Species-specific variation is also shown, including PTI-associated ROS-mediated basal resistance in Arabidopsis and delayed, non-classical HR resistance in cowpea.

Overall, antioxidant enzyme regulation is a decisive layer of plant–RKN immunity (Figure 3). Resistant hosts generally maintain a redox environment in which SOD-driven H_2_O_2_ production is not fully neutralized by CAT or APX, allowing H_2_O_2_ to activate SA signaling, HR, phenylpropanoid metabolism, callose deposition, and lignification. Susceptible hosts, in contrast, often co-induce ROS-generating and ROS-scavenging enzymes, producing a buffered redox state that supports giant cell development. However, species-specific exceptions such as cowpea demonstrate that ROS-based resistance is not universal in form, even when redox signaling remains an important component of the plant–nematode interaction.

## 5. Effector-Mediated Rewiring of Host ROS Networks During Root-Knot Nematode Parasitism

Successful parasitism by RKNs requires more than passive tolerance of plant-derived ROS. Because RKNs are obligate biotrophs that must establish and maintain living giant cells, they actively suppress, redirect, and buffer host ROS signaling to avoid HR-associated cell death while preserving a redox environment permissive for feeding site development. A growing body of evidence shows that RKNs and related sedentary plant-parasitic nematodes secrete effectors that target multiple nodes of the host ROS network, including upstream PTI signaling, Ca^2+^-dependent RBOH activation, plastid redox metabolism, H_2_O_2_ scavenging, and SA-dependent defense regulation (Table 1).

### 5.1. Suppression of Upstream PTI and RBOH-Dependent ROS Production

Several nematode effectors act before or during ROS generation by interfering with immune signaling upstream of RBOH activation. Mi-CRT, a calreticulin secreted from the subventral gland cells of *M. incognita*, chelates extracellular Ca^2+^ in the apoplast and thereby limits the Ca^2+^ influx required for RBOHD activation. Because Ca^2+^ binding to RBOH EF-hand domains is essential for the PTI-associated oxidative burst, Mi-CRT suppresses ROS production as well as downstream immune outputs such as defense gene expression and callose deposition [18,72]. RNA interference-mediated knockdown of Mi-CRT reduces nematode infection and restores higher RBOH-dependent ROS accumulation in host roots, confirming its role as a virulence factor that weakens early immune signaling.

A similar upstream suppression strategy is represented by GrCEP12, a ubiquitin carboxyl extension protein from the cyst nematode *Globodera rostochiensis* [73,74]. GrCEP12 suppresses flg22-induced PTI responses, including ROS burst, MAPK activation, callose deposition, and PTI marker gene expression, apparently acting downstream of receptor perception but upstream of RBOH-mediated ROS production [5]. Other effectors also modulate early immune and ROS responses. MiPDCD6 from *M. incognita* suppresses SA-mediated immunity and thereby indirectly reduces ROS accumulation, whereas MgMO289 from *M. graminicola* interacts with rice OsHPP04 to activate host O_2_•^−^-scavenging capacity [8,16,18,64,75].

### 5.2. MjTTL5-Mediated Exploitation of the Plastid Ferredoxin–Thioredoxin System

MjTTL5 is one of the best-characterized ROS-suppressing RKN effectors. It is a 151-amino acid transthyretin-like protein secreted from the subventral esophageal glands of *M. javanica* during early parasitism, precisely when giant cell initiation and immune suppression are required [7]. Although MjTTL5 lacks a canonical chloroplast transit peptide, it localizes to host plastids through a non-canonical import mechanism, resembling strategies used by some bacterial effectors that target chloroplast immunity.

Mechanistically, MjTTL5 interacts with the catalytic subunit of *Arabidopsis* ferredoxin–thioredoxin reductase, AtFTRc, a central component of the plastid redox regulatory system. Yeast two-hybrid assays, co-immunoprecipitation, and bimolecular fluorescence complementation confirmed the MjTTL5–AtFTRc interaction in planta [7]. Functionally, this interaction greatly enhances the H_2_O_2_-scavenging capacity of host protein extracts: addition of purified MjTTL5 to *Arabidopsis* protein extracts nearly eliminates H_2_O_2_ within minutes, whereas plant extracts alone remove only a fraction of the available H_2_O_2_. Thus, MjTTL5 does not act as an antioxidant enzyme by itself; rather, it hijacks a host plastid redox hub to increase endogenous ROS-scavenging activity.

Genetic evidence supports the virulence function of this interaction. *Arabidopsis* plants expressing MjTTL5 show increased susceptibility to *M. javanica*, *M. incognita*, and *Radopholus similis*, whereas in planta RNAi-mediated silencing of MjTTL5 reduces nematode reproduction by approximately 34–38% [7]. Conversely, *atftrc* loss-of-function mutants show enhanced resistance to *M. javanica*, indicating that AtFTRc acts as a host susceptibility factor co-opted by the nematode. The high conservation of TTL5 homologs among *Meloidogyne* species and other plant-parasitic nematodes, but not free-living nematodes, suggests that TTL5-mediated manipulation of host antioxidant systems is an evolutionarily conserved parasitism strategy.

### 5.3. CATLe-Mediated Direct Decomposition of H_2_O_2_

CATLe represents a distinct ROS-suppression mechanism because it directly degrades H_2_O_2_ rather than activating host antioxidant machinery. CATLe is a catalase domain-containing effector from *M. incognita* that is expressed in subventral gland cells, upregulated during the early infection window of 24–72 h post-infection, and secreted into developing feeding sites [76]. Biochemical assays indicate that CATLe possesses strong catalase activity, approximately 42,500 U/mg protein, enabling it to catalytically decompose H_2_O_2_ at the plant–nematode interface.

Transient expression of CATLe in *Nicotiana benthamiana* suppresses flg22-induced ROS production, demonstrating its ability to dampen PTI-associated oxidative responses. Conversely, dsRNA-mediated silencing of CATLe in infective juveniles increases H_2_O_2_ levels in infected tomato radicles at 24 h post-inoculation and reduces gall number, gall size, and egg mass production [76]. CATLe orthologs are conserved across major *Meloidogyne* species, suggesting that direct enzymatic detoxification of host H_2_O_2_ is a broadly conserved RKN virulence strategy [16,76]. The CATLe phenotype supports an upper oxidative constraint on RKN parasitism but does not establish a lower H_2_O_2_ requirement for giant-cell development.

### 5.4. Mj-NEROSs and Manipulation of Plastid-Derived ROS

RKNs also target plastid electron transport to reduce organelle-derived ROS. Mj-NEROSs, a *M. javanica* nematode effector ROS suppressor, localizes to plastids and interacts with the Rieske iron–sulfur protein, a component of the cytochrome b_6_f complex involved in photosynthetic electron transport [16,28]. By interacting with Rieske ISP, Mj-NEROSs alters electron transport dynamics and reduces plastid-derived ROS production.

Functionally, Mj-NEROSs suppresses flg22-induced ROS accumulation, INF1-triggered cell death, and callose deposition in *N. benthamiana* [28]. *Arabidopsis isp* mutants show enhanced susceptibility to *M. javanica*, supporting the conclusion that plastid-derived ROS contribute to nematode defense and that Mj-NEROSs promotes parasitism by weakening this defense layer [28,43]. Together with MjTTL5, Mj-NEROSs highlights plastids as major targets of RKN effectors and demonstrates that nematodes manipulate not only apoplastic RBOH-derived ROS but also intracellular organelle-based redox signaling.

### 5.5. Suppression of SA-Dependent ROS Amplification by Chorismate Mutase

In addition to directly suppressing ROS production or accelerating H_2_O_2_ detoxification, RKNs interfere with hormonal pathways that regulate antioxidant enzymes. Chorismate mutase II, a secreted effector from *M. incognita*, diverts chorismate away from SA biosynthesis toward prephenate metabolism [8,18,77]. Because SA-dependent immune signaling is associated with changes in antioxidant capacity and H_2_O_2_ homeostasis, CMII-mediated reduction in SA may weaken defense-associated redox regulation during compatible interactions.

This mechanism is consistent with transcriptomic and pharmacological evidence. In susceptible tomato roots, *M. incognita* suppresses SA-responsive genes such as *PR-1*, *PR-2*, *PR-4b*, and *PR-5* during early infection [18]. Pharmacological reduction in SA levels with paclobutrazol increases nematode infection, whereas methyl salicylate treatment reduces galling and nematode reproduction by approximately 50%. CMII-like chorismate mutases have also been identified in other endoparasitic nematodes, suggesting that suppression of SA-dependent ROS amplification through chorismate diversion may be a conserved strategy among plant-parasitic nematodes [8,18].

### 5.6. Nematode Intrinsic Antioxidant Systems as Self-Protection Mechanisms

RKNs not only manipulate host ROS networks but also deploy their own antioxidant defenses to survive oxidative stress at the infection interface. Peroxiredoxins (PRXs), non-heme PODs that use TRX to reduce H_2_O_2_, are present in the hypodermis and cuticle-associated tissues of *M. incognita* J2s, where they are positioned to detoxify plant-derived apoplastic ROS [5,78]. RNAi-mediated knockdown of *M. incognita* PRXs reduces root galling, demonstrating that nematode-intrinsic antioxidant capacity is important for successful parasitism.

Transcriptome analyses further show that nematodes activate antioxidant and stress-protection genes when confronting resistant hosts. During *Mi-1.2*-mediated resistance in tomato, *M. incognita* upregulates genes encoding CAT, SOD, GPX, GSTs, PRXs, and C-type lectins, several of which are associated with ROS detoxification or suppression of host ROS production [8]. The induction of autophagy-related proteins and peptidases during resistant interactions may reflect starvation and stress responses associated with failed giant cell maintenance and ROS-mediated feeding site collapse.

Collectively, ROS-targeting effectors reveal that RKNs suppress plant immunity through a multilayered redox strategy (Table 1). Mi-CRT may attenuate Ca^2+^-dependent immune signaling, whereas GrCEP12 suppresses upstream PTI outputs in a cyst-nematode system; MjTTL5 and Mj-NEROSs target plastid redox systems; CATLe directly degrades H_2_O_2_; CMII suppresses SA-dependent ROS amplification; and nematode antioxidant enzymes protect the parasite from residual host ROS. These mechanisms demonstrate that compatibility depends on multilayered redox manipulation rather than simple ROS elimination. Collectively, these effectors can shift host redox conditions toward compatibility. However, current evidence does not demonstrate that RKNs sense H_2_O_2_ levels or actively maintain a fixed redox setpoint.

## 6. The Redox Paradox: RBOH-Derived ROS as Defense Signals and Potential Feeding-Site Stabilizers

**Figure 4:**
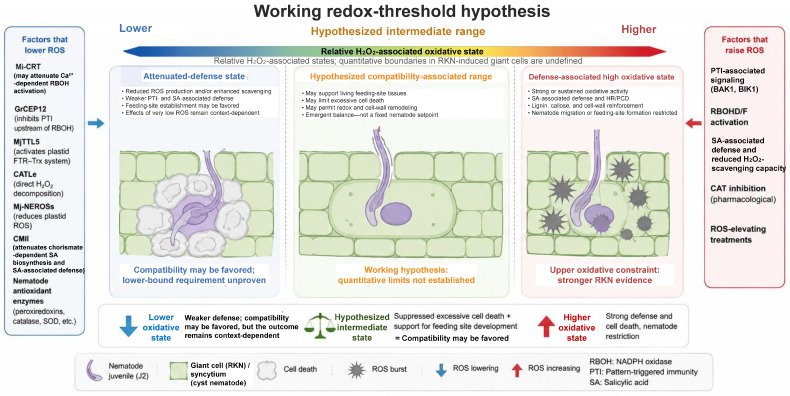
Working redox-threshold hypothesis for ROS regulation in plant–nematode interactions. The low, intermediate, and high oxidative states shown are relative and do not represent defined H_2_O_2_ concentrations. Reduced ROS production or enhanced scavenging can weaken basal immunity and favor compatibility, although the consequences of very low ROS remain context-dependent. An intermediate compatibility-associated redox range is hypothesized to support living feeding-site tissues while limiting excessive cell death, but its quantitative boundaries have not been established. Strong or sustained oxidative activity is associated with SA-dependent defense, HR-associated cell death, structural reinforcement, and nematode restriction. Evidence for an upper oxidative constraint is comparatively strong in RKN systems, whereas evidence for a lower boundary derives mainly from the *Arabidopsis–Heterodera schachtii* syncytium system. Neither direct quantitative H_2_O_2_ measurements nor evidence for a fixed redox setpoint or an RKN H_2_O_2_-sensing mechanism is currently available for RKN-induced giant cells.

A central paradox in plant–nematode redox biology is that RBOH-derived ROS can contribute to immunity while, under particular conditions, also supporting the survival of the living tissues required for sedentary nematode feeding. The evidence for these opposing functions is not equally strong across nematode systems. Direct genetic evidence that RBOH-derived ROS promote feeding-site establishment by restricting excessive cell death comes primarily from the *Arabidopsis thaliana–Heterodera schachtii* interaction. In RKN systems, by contrast, the available evidence more clearly establishes a defense-promoting role for ROS and an upper oxidative constraint associated with impaired parasitism. Whether RKN-induced giant cells also require a minimum level of ROS for their development or maintenance remains unresolved. To address both the evidentiary limitations and the mechanistic asymmetry of the proposed model, the following subsections first evaluate direct genetic evidence from the cyst-nematode system and the distinct genetic, cytological, pharmacological, and effector-based evidence available from RKN systems. They then examine spatial, developmental, and compartment-specific redox regulation and assess whether any evidence supports nematode-intrinsic sensing or active maintenance of a compatibility-associated redox range (Figure 4).

### 6.1. Foundational Genetic Evidence from the H. schachtii–Arabidopsis Interaction

The strongest direct evidence for a pro-compatibility function of host RBOH-derived ROS comes from the interaction between *A. thaliana* and the beet cyst nematode *H. schachtii*. Siddique et al. [17] showed that disruption of *RBOHD*, with the strongest phenotype observed in the *rbohD rbohF* double mutant, reduced nematode development and syncytium formation. In the double mutant, this reduction was accompanied by extensive cell death in tissues surrounding the developing feeding sites. These findings indicate that RBOHD/F-derived ROS restrict the spatial spread of cell death and thereby preserve the living tissue required for syncytium establishment. In this system, therefore, host ROS function not only as immune signals but also as negative regulators of uncontrolled cell death that can facilitate nematode infection. Subsequent work linked RBOHD/F-dependent ROS to cyst-nematode-induced reprogramming of host indole metabolism, providing an additional mechanistic route through which NADPH oxidase activity can promote infection [38].

This pro-compatibility function is not shared uniformly by all RBOH isoforms. *AtRBOHB* is strongly downregulated in developing syncytia, whereas its overexpression enhances resistance to *H. schachtii* [79]. Thus, RBOHD/F and RBOHB can have contrasting effects during cyst-nematode infection. These findings argue against treating total RBOH activity as a single functional unit and instead support isoform- and tissue-specific regulation of ROS during feeding-site formation.

### 6.2. Evidence from RKN Systems: Defense-Promoting ROS and Residual Oxidative Activity

In RKN interactions, the clearest genetic evidence supports a defense-promoting function of RBOH-derived ROS. *Arabidopsis* mutants compromised in BAK1-, BIK1-, or RBOHD/F-dependent signaling exhibit increased susceptibility to *M. incognita*, indicating that PTI-associated ROS production contributes to basal RKN resistance [5]. This finding differs from the pro-parasitic role of RBOHD/F observed during *H. schachtii* infection and emphasizes that the biological effect of a given RBOH isoform depends on the nematode species, feeding-site type, infection stage, and surrounding cellular context.

Cytological studies nevertheless indicate that compatible RKN interactions are not characterized by a complete absence of oxidative activity. During tomato infection by *M. incognita*, an early oxidative response occurs in both compatible and incompatible interactions. The signal is subsequently sustained and expanded in incompatible interactions but attenuated in compatible interactions [6]. H_2_O_2_-associated deposits have also been detected at plasma-membrane and cell-wall regions near developing feeding sites. These observations indicate that compatible infection involves spatial and temporal remodeling of oxidative activity rather than its complete elimination. However, the persistence of H_2_O_2_ at or near giant cells does not by itself demonstrate that H_2_O_2_ is required for giant-cell development.

### 6.3. Pharmacological and Effector-Based Evidence for an Upper Oxidative Constraint

Pharmacological manipulation of ROS production and scavenging has been associated with changes in RKN infection outcomes. In susceptible tomato, treatments expected to reduce ROS production or enhance ROS removal were generally associated with increased infection, whereas treatments expected to elevate ROS were associated with reduced galling or nematode reproduction [18]. These findings are consistent with a defense-promoting role for ROS. However, the compounds used may have off-target effects, and the experiments were not designed as calibrated H_2_O_2_ concentration–response analyses. They therefore do not establish a quantitatively defined compatibility window or a lower H_2_O_2_ boundary.

Effector studies provide stronger evidence that elevated H_2_O_2_ is detrimental to RKN parasitism. The *M. incognita* catalase-like effector CATLe directly degrades H_2_O_2_, and suppression of *CATLe* expression increases H_2_O_2_ accumulation while reducing gall formation and nematode reproduction [76]. Similarly, MjTTL5 enhances host H_2_O_2_-scavenging capacity through the plastid ferredoxin–thioredoxin system, whereas Mj-NEROSs alters plastid electron transport and reduces the associated ROS output [7,28]. Collectively, these studies support the conclusion that nematode-mediated suppression or detoxification of host ROS promotes parasitism in the tested systems. They support the existence of an upper oxidative constraint but do not demonstrate that excessive ROS depletion impairs RKN feeding-site development.

### 6.4. Plausible Developmental Functions of Controlled ROS at Feeding Sites

Controlled ROS may participate in cellular processes relevant to giant-cell and syncytium development, but most proposed developmental functions remain inferential. Giant-cell formation requires extensive cell-wall remodeling, cellular expansion, altered plasmodesmatal connectivity, and sustained metabolic reprogramming. H_2_O_2_ can serve as a substrate for class III peroxidases, which may promote either cell-wall stiffening through extensin cross-linking and lignin polymerization or wall loosening through radical-dependent chemistry, depending on the enzyme isoform, available substrates, transition-metal availability, and local redox conditions [80]. The localization of H_2_O_2_-associated deposits near giant-cell walls is therefore compatible with a role in cell-wall remodeling, but this observation does not establish causality.

More speculative links between ROS and ion-channel regulation, cell-cycle re-entry, or endoreduplication should be regarded as hypotheses rather than as established components of feeding-site biology. Direct experiments have not yet shown that manipulating H_2_O_2_ specifically within giant cells alters these developmental processes independently of immune signaling or oxidative injury.

### 6.5. Spatial, Temporal, and Isoform-Specific Redox Regulation

The apparent paradox becomes more comprehensible when ROS are considered as compartmentalized and isoform-specific signals rather than as a homogeneous pool. In the cyst-nematode system, RBOHD/F-derived ROS in tissues surrounding the syncytium can restrict the spread of cell death, whereas RBOHB appears to contribute to resistance and is downregulated within developing feeding sites. In RKN interactions, different effectors target distinct components of host redox regulation. Mi-CRT suppresses apoplastic basal immune responses and, as a Ca^2+^-binding protein, may influence Ca^2+^-dependent ROS signaling; however, direct inhibition of RBOH activation by Mi-CRT has not been demonstrated [72]. MjTTL5 enhances plastid-associated H_2_O_2_ scavenging, CATLe directly removes H_2_O_2_, and Mj-NEROSs alters plastid ROS production.

This compartment-specific manipulation demonstrates that nematodes remodel multiple ROS pools through different mechanisms. However, it does not prove that residual ROS are actively maintained at an optimal concentration. Residual oxidative activity may arise from incomplete effector-mediated suppression, continued host RBOH activity, organelle-derived ROS, or stage-dependent changes in host and nematode gene expression. Whether nematodes can sense these residual redox states or actively compensate for excessive ROS depletion by increasing host- or nematode-derived ROS is considered in the following subsection.

### 6.6. Nematode-Intrinsic Redox Responsiveness and the Mechanistic Asymmetry of the Proposed Compatibility Window

Evidence for nematode-intrinsic redox responsiveness is emerging, but direct molecular sensing of host-derived H_2_O_2_ has not been demonstrated in RKNs. *Meloidogyne incognita* possesses *MiDaf16-like1* and *MiSkn1-like1*, which encode homologs of the redox-responsive transcriptional regulators DAF-16/FOXO and SKN-1/Nrf2, respectively. Their expression is modulated by oxidative stress, and host-induced silencing of these genes impairs nematode infection and reproduction [81]. In *Caenorhabditis elegans*, oxidative stress promotes PMK-1-dependent nuclear accumulation and activation of SKN-1 [82]. The corresponding *M. incognita* proteins should therefore be interpreted as downstream components of a conserved oxidative-stress response network rather than as direct H_2_O_2_ sensors. Similarly, Clade B PRXs expressed in the hypodermis and pseudocoelom of *M. incognita* detoxify peroxides and contribute to successful parasitism [78]. Their localization and biochemical activity make them plausible participants in peroxide-responsive redox signaling, but such a signaling role has not been experimentally demonstrated in RKNs.

The proposed compatibility-window model also remains mechanistically asymmetric. Several RKN effectors reduce host ROS production or enhance ROS removal, including MjTTL5, CATLe, and Mj-NEROSs. Other effectors, such as Mi-CRT, may influence host redox homeostasis more indirectly by suppressing basal immunity. However, no RKN effector has been shown to increase host ROS in response to experimentally reduced ROS levels. Any apparent lower boundary, if it exists, may therefore emerge passively from residual host RBOH activity, incomplete effector-mediated suppression, organelle-derived ROS, or stage-dependent changes in host and nematode gene expression rather than from an active nematode-controlled homeostatic mechanism.

This asymmetry means that the Goldilocks model should not currently be interpreted as evidence that RKNs sense H_2_O_2_ concentrations and actively maintain an optimal redox state. A more conservative interpretation is that compatible infection may occur within an emergent redox range generated by the balance between immune-associated ROS production and nematode-mediated ROS attenuation. Whether RKNs possess secreted enzymes or effectors that actively increase host- or nematode-derived ROS under strongly reducing conditions remains unknown.

### 6.7. Quantitative Definition of the Compatibility Window and Biosensor-Based Experimental Priorities

#### 6.7.1. Quantitative Status of the Working Redox-Threshold Hypothesis

No study has directly determined the absolute H_2_O_2_ concentration within developing RKN-induced giant cells or in the apoplast immediately surrounding a feeding site. Current plant–RKN studies have relied mainly on bulk-root biochemical measurements, fluorescence-based assays, histochemical localization using CeCl_3_ or DAB, and changes in the activities or expression of ROS-producing and ROS-scavenging enzymes [6,31]. These approaches reveal relative spatial and temporal differences in oxidative activity but cannot resolve absolute, cell-type-specific H_2_O_2_ concentrations. DCFH-based fluorescence is additionally responsive to multiple oxidizing species and should not be interpreted as a direct quantitative measurement of H_2_O_2_.

Consequently, neither the upper nor the lower H_2_O_2_ boundary of the proposed compatibility-associated redox range has been defined. Concentrations reported after exogenous H_2_O_2_ application or in non-nematode experimental systems cannot be transferred directly to RKN feeding sites because the effective redox signal depends on subcellular location, pH, antioxidant capacity, exposure duration, diffusion, and the rates of H_2_O_2_ production and removal. Assigning a provisional range such as 0.1–5 µM to compatibility and 10–100 µM to defense would therefore imply a level of quantitative support that is not currently available.

Available evidence supports an upper oxidative constraint more strongly than a lower boundary. Pharmacological elevation of ROS and silencing of the H_2_O_2_-degrading effector CATLe are associated with reduced galling or nematode reproduction, whereas incompatible interactions commonly exhibit sustained oxidative activity. By contrast, evidence for a lower boundary is largely qualitative and derives mainly from the *Arabidopsis–Heterodera schachtii* system, in which disruption of RBOHD/F promotes extensive cell death and impairs syncytium formation. Whether a comparable lower-redox constraint exists in RKN-induced giant cells remains unknown. The Goldilocks model should therefore be regarded as a working redox-threshold hypothesis rather than as an established quantitative model or an actively maintained nematode homeostatic mechanism.

#### 6.7.2. Genetically Encoded Biosensors for Testing the Hypothesis

Genetically encoded fluorescent biosensors provide a promising means of resolving H_2_O_2_-associated redox dynamics at single-cell and subcellular resolution. HyPer sensors contain a circularly permuted fluorescent protein linked to an OxyR-derived H_2_O_2_-responsive domain and generate a ratiometric fluorescence response following sensor oxidation [83]. HyPer7 provides substantially improved sensitivity, response kinetics, brightness, and pH stability relative to earlier HyPer variants [84]. However, its performance in nematode-infected plant tissues and in strongly oxidizing compartments such as the apoplast must be validated experimentally before quantitative interpretation.

The roGFP2-Orp1 sensor provides a complementary readout. In this construct, the yeast POD Orp1 functions as an H_2_O_2_-responsive relay that transfers oxidative equivalents to roGFP2, producing a ratiometric signal [85]. This signal reflects H_2_O_2_-dependent oxidation integrated with the local capacity to reduce the sensor; it does not directly report the glutathione redox potential. Grx1-roGFP2 is better suited for monitoring *E*_GSH_ and can therefore provide complementary information on cellular redox buffering [86]. Plant studies using organelle-targeted roGFP2-Orp1 and Grx1-roGFP2 have demonstrated that pattern-triggered immune responses generate distinct, compartment-specific oxidative signatures rather than a uniform cellular ROS response [87].

Importantly, the oxidation ratios of HyPer7 and roGFP2-Orp1 should not be equated automatically with absolute H_2_O_2_ concentrations. Sensor output depends on oxidation kinetics, enzymatic reduction, pH, expression level, compartmental environment, and the available dynamic range. Quantitative comparisons among cell types, infection stages, and genotypes will therefore require in planta determination of fully reduced and oxidized sensor states, pH controls where appropriate, and validation that the sensor remains responsive and unsaturated in the target compartment.

A rigorous experimental design would express these sensors using validated giant-cell- or feeding-site-enriched promoters and compare developing giant cells, adjacent vascular cells, and surrounding cortical tissues across infection stages. Corresponding infection-site cells should be examined during incompatible interactions because fully developed giant cells may not form. Cytosolic and nuclear sensors could be complemented by sensors targeted to plastids, mitochondria, peroxisomes, and—following compartment-specific validation—the apoplast. Time-resolved imaging should be combined with inducible or cell-type-specific manipulation of RBOH, CAT, APX, and ROS-targeting nematode effectors.

Such experiments should determine whether: (i) compatible giant cells exhibit a reproducible redox state distinct from that of adjacent cells; (ii) experimentally increasing oxidative activity above this state restricts feeding-site development; (iii) reducing oxidative activity impairs giant-cell initiation or maintenance; and (iv) the relevant redox boundaries vary with infection stage, host genotype, nematode species, and subcellular compartment. These measurements would provide the first direct experimental test of the quantitative predictions of the Goldilocks hypothesis.

## 7. ROS-Dependent Downstream Defense and Systemic Signaling in Plant–Root-Knot Nematode Interactions

ROS, particularly H_2_O_2_, function as central hubs that connect local root immune activation to structural defense, hormonal regulation, and whole-plant systemic resistance during RKN interactions. Once generated at the infection site, ROS do not act as isolated toxic molecules; instead, they are integrated into defense networks involving SA, JA, BRs, lignin, callose, suberin, electrical signaling, and root-to-shoot communication. The effectiveness of ROS-mediated resistance therefore depends on whether the initial oxidative burst is successfully translated into local and systemic defense outputs.

### 7.1. ROS as Triggers of Cell Wall Reinforcement

H_2_O_2_ generated during the oxidative burst activates several cell wall-based defense mechanisms that physically restrict nematode invasion and feeding site development. One major route is lignification. PODs, often measured as activity, use H_2_O_2_ to oxidize monolignols, thereby promoting lignin polymerization in the cell wall. This lignified barrier mechanically impedes nematode migration and reduces the effectiveness of nematode-derived cell wall-degrading enzymes [45]. In resistant sweetpotato and carrot, elevated H_2_O_2_ accumulation is closely associated with enhanced phenylpropanoid metabolism, increased total phenolic content, and lignin deposition, supporting the view that ROS act upstream of structural defense activation [45,46].

H_2_O_2_ also promotes callose deposition at cell walls surrounding infection sites and at plasmodesmata. This response may limit both the symplastic movement of nematode effectors and the nutrient flux required for giant cell establishment. In resistant carrot, callose deposition appears before strong lignin accumulation, suggesting that callose functions as an early barrier that is later reinforced by lignification [46]. ROS-dependent suberin deposition may provide an additional layer of defense by reinforcing root tissues and restricting nematode migration, although its precise contribution to RKN resistance remains less clearly defined than that of lignin and callose.

### 7.2. Context-Dependent SA–H_2_O_2_ Crosstalk in Incompatible Interactions

SA and H_2_O_2_ participate in reciprocal and context-dependent crosstalk during plant immunity. H_2_O_2_ can influence SA-dependent defense signaling, whereas SA can reshape H_2_O_2_ homeostasis through changes in antioxidant-enzyme activity, abundance, turnover, reductant availability, and broader redox regulation. Direct inhibition of CAT by SA may contribute under particular biochemical conditions, and APX is a comparatively sensitive in vitro target of SA; however, neither mechanism has been established as the universal driver of H_2_O_2_ accumulation in nematode-infected roots, as discussed in Section 4.3.

In tomato, *Mi-1.2*-mediated resistance is closely associated with SA signaling. SA activates NPR1-dependent transcriptional programs, including TGA-regulated PR genes with defense-related functions. Conversely, during compatible interactions, *M. incognita* suppresses SA-responsive immunity. The nematode chorismate mutase effector CMII may divert chorismate away from SA biosynthesis toward prephenate metabolism, thereby weakening SA-dependent immune signaling and altering redox homeostasis; however, CMII has not been shown to maintain CAT activity through a defined direct mechanism [8,18]. Pharmacological evidence supports a protective role for SA signaling: methyl salicylate treatment reduces nematode infection, whereas reduction in SA levels with paclobutrazol increases susceptibility [18].

### 7.3. Jasmonate–ROS Crosstalk: Pharmacological Limitations, Defense Functions, and Possible Roles in Gall Development

#### 7.3.1. Limitations of Pharmacological Evidence

Pharmacological evidence for JA function in plant–RKN interactions must be interpreted cautiously. Molinari et al. [18] examined tomato plants treated with compounds that affect SA, JA/ethylene, Ca^2+^, and ROS-associated pathways. Treatments described as reducing JA/ET-associated signaling did not significantly alter *M. incognita* infection. Thus, this study does not demonstrate that salicylhydroxamic acid (SHAM) reduces RKN infection or establish a JA-specific contribution to basal resistance.

SHAM is commonly used as a lipoxygenase inhibitor, but it is not specific to the JA-producing branch of oxylipin metabolism. Depending on its concentration and the experimental system, SHAM can affect multiple lipoxygenases, mitochondrial alternative oxidase, and peroxidase activity. Its effects may therefore arise from changes in JA, 12-oxo-phytodienoic acid (OPDA), other oxylipins, mitochondrial metabolism, H_2_O_2_ turnover, or cell-wall-associated peroxidase reactions. SHAM-derived phenotypes should consequently be regarded as evidence for perturbation of a broader redox–oxylipin network rather than as direct evidence for JA depletion. Definitive analysis requires JA- and OPDA-biosynthetic mutants, jasmonoyl-isoleucine (JA-Ile) signaling mutants, direct quantification of individual oxylipins, and controls for alternative oxidase and peroxidase activity. In canonical jasmonate signaling, JA-Ile promotes formation of the COI1–JAZ co-receptor complex, leading to SCF^COI1-dependent degradation of JAZ repressors and release of MYC-family transcription factors. This module can activate defense-related and developmental transcriptional programs. However, its cell-type-specific contribution to resistance or feeding-site development during RKN infection remains insufficiently resolved and should not be inferred solely from exogenous JA treatments.

#### 7.3.2. Genetic and Physiological Evidence for JA- and OPDA-Mediated Defense

Evidence from several pathosystems indicates that JA-associated signaling can enhance resistance to RKNs. In tomato, the JA-deficient *spr2* mutant was more susceptible to RKN infection, whereas activation of the systemin/JA pathway and exogenous JA treatment enhanced defense-related responses [88]. Exogenous JA also reduced *M. incognita* egg-mass production, and pharmacological and gene-silencing experiments implicated NO and proteinase inhibitor 2 in this response [89]. In rice, JA biosynthesis was required for systemically induced resistance against *M. graminicola* following treatment with JA- or ET-related inducers [90].

The *Arabidopsis–M. hapla* interaction further demonstrates that the oxylipin pathway cannot be represented as a single linear JA signal. Mutants deficient in both OPDA and JA production were hypersusceptible, whereas *opr3*, which accumulates OPDA but is impaired in its conversion to JA, showed wild-type levels of galling. Mutations affecting downstream JA biosynthesis or perception also did not reproduce the hypersusceptibility of mutants deficient in upstream OPDA production [91]. These findings support a protective function for OPDA or an OPDA-associated pathway that is at least partly distinct from canonical JA-Ile–COI1 signaling.

Collectively, these studies show that JA-associated pathways frequently promote defense rather than susceptibility. However, the relevant signal may differ among JA, JA-Ile, OPDA, and other oxylipins, and the outcome varies with plant species, nematode species, tissue, developmental stage, and treatment regime.

#### 7.3.3. SA–JA Crosstalk in RKN Interactions

SA and JA signaling frequently antagonize one another in plant immunity, but this relationship is conditional rather than universal. Its outcome depends on hormone concentration, timing, tissue, attacker identity, and the activation state of other signaling pathways [92,93]. Consequently, suppression of SA during compatible RKN infection should not automatically be interpreted as evidence of enhanced JA signaling.

In susceptible tomato roots, Molinari et al. [18] observed downregulation of several SA-responsive *PR* genes, whereas the JA/ethylene-responsive genes examined were not induced. This result supports suppression of SA-associated immunity but does not demonstrate reciprocal activation of JA. In another biological context, *Trichoderma*-induced resistance against *M. incognita* involved temporally coordinated SA- and JA-regulated responses, with an early SA-associated phase followed by a later JA-associated phase [94]. This temporal transition illustrates that SA and JA can act sequentially rather than as a simple mutually exclusive switch.

The consequences of SA–JA crosstalk for ROS homeostasis during RKN infection remain insufficiently resolved. In particular, there is no direct evidence that SA suppression necessarily increases JA accumulation, that JA activation directly lowers or raises RBOH-derived ROS, or that SA–JA antagonism controls CAT activity in infected roots through a simple linear mechanism. Resolving these relationships will require simultaneous measurements of SA, JA, JA-Ile, and OPDA together with cell-type-resolved ROS dynamics and genetic perturbation of both hormone pathways.

#### 7.3.4. Cell-Wall Remodeling, Gall Expansion, and JA–ROS Research Gaps

The development of giant cells and surrounding gall tissue requires extensive cell expansion and cell-wall remodeling. Transcriptomic analyses of nematode-induced giant cells and galls have identified altered expression of expansins, xyloglucan endotransglucosylases/hydrolases, pectin-modifying enzymes, and other wall-associated proteins [95]. These observations provide a biological basis for proposing that hormone-regulated cell-wall plasticity contributes to feeding-site development.

However, increased expression of cell-wall-remodeling genes does not by itself demonstrate that JA induces these genes, that their induction promotes susceptibility, or that the process is independent of ROS. Direct evidence linking endogenous JA or JA-Ile to expansin-dependent gall expansion is currently limited. Moreover, the protective effects of JA and OPDA observed in tomato, rice, and *Arabidopsis* argue against a universal model in which JA functions primarily as a susceptibility hormone. JA-dependent facilitation of gall expansion should therefore be treated as a testable hypothesis whose validity may be restricted to particular tissues, developmental stages, or host–nematode combinations.

Oxylipin and redox metabolism nevertheless have several potential points of interaction. OPDA is a reactive electrophilic molecule that can influence GSH-associated metabolism, and JA-dependent defense against RKNs can intersect with NO signaling [89,96]. Such interactions could indirectly modify H_2_O_2_ production or scavenging. However, direct JA-dependent regulation of RBOH activity, an RKN-specific JAZ–WRKY–RBOH module, and an H_2_O_2_–LOX feedback loop has not been demonstrated in infected roots. These proposed connections should therefore be considered mechanistic hypotheses rather than established components of RKN defense.

Future studies should combine JA-, OPDA-, and JA-Ile-pathway mutants with direct oxylipin quantification, genetically encoded ROS reporters, cell-specific transcriptomics, and functional manipulation of expansins and other wall-remodeling genes. Such experiments are required to determine whether JA–ROS crosstalk primarily promotes defense, contributes to feeding-site development, or performs temporally distinct functions during different phases of RKN infection.

### 7.4. BR–RBOH1–MAPK Signaling as a Resistance-Promoting Pathway

BRs provide another hormonal route through which ROS production is linked to nematode resistance. In tomato, BR signaling positively regulates resistance against *M. incognita* through an RBOH1-dependent MAPK pathway. Exogenous BR application reduces susceptibility, whereas BR-deficient or BR-insensitive mutants show increased susceptibility [35]. BR-induced resistance is accompanied by increased apoplastic ROS accumulation, elevated RBOH1 transcript levels, and activation of MPK1/2 and MPK3. Importantly, silencing *RBOH1* abolishes BR-induced MAPK activation, indicating that RBOH1 functions as the proximal ROS-producing enzyme in this pathway. This BR–RBOH1–MAPK module provides a mechanistic link between hormone perception, oxidative burst activation, and downstream defense signaling.

### 7.5. Systemic ROS–Electrical Signaling and Root-to-Shoot Defense Coordination

Beyond local defense at the infection site, RKN attack can trigger systemic root-to-shoot signaling that coordinates whole-plant immunity. In tomato, *M. incognita* infestation induces electrical signals and an RBOH1-dependent ROS wave that propagate from infected roots through the stem to aerial tissues. Genetic and grafting experiments showed that GLR3.5 and RBOH1 are required for efficient systemic electrical and ROS signal propagation. Disruption of either component reduced JA accumulation and MPK1/2 activation in leaves and compromised resistance to RKN infection [59]. These findings support a tomato systemic defense circuit in which GLR3.5-dependent electrical signaling and RBOH1-dependent ROS propagation promote shoot JA signaling and contribute to whole-plant resistance. This RKN-induced systemic pathway should be distinguished from the GLR3.3/GLR3.6-mediated wound-signaling pathway characterized in *Arabidopsis*.

### 7.6. Systemic Consequences of Local Redox Manipulation

The existence of root-to-shoot ROS and electrical signaling helps explain why local redox manipulation can have unexpected whole-plant consequences. Treatments or nematode effectors that suppress ROS at the infection site may not only affect local H_2_O_2_ accumulation but also weaken systemic JA signaling and distal defense readiness. Conversely, excessive local ROS may trigger strong defense but also risk tissue damage or maladaptive systemic responses. Moreover, RKN infection may negatively modulate aboveground immune responsiveness, reducing the ability of leaves to respond effectively to foliar pathogens, suggesting that nematode-induced systemic signaling can involve both defense activation and immune trade-offs [16,18].

**Figure 5:**
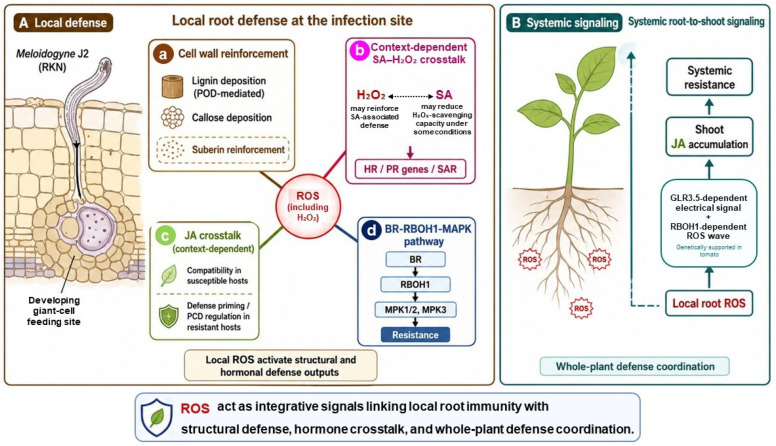
ROS-dependent downstream defense and systemic signaling in plant–root-knot nematode interactions. (**A**) Local defense at the infection site. Following invasion by *Meloidogyne* second-stage juveniles (J2s), H_2_O_2_/ROS function as central signaling molecules that activate multiple local defense outputs in infected roots. ROS promote cell wall reinforcement through POD-mediated lignin deposition, callose accumulation, and possible suberin reinforcement. H_2_O_2_ interacts with salicylic acid (SA)-dependent immunity through a context-dependent regulatory network involving changes in antioxidant-enzyme activity, abundance, and redox signaling. Direct inhibition of CAT or APX by SA may contribute under particular biochemical conditions, but neither mechanism has been established as the universal cause of H_2_O_2_ accumulation in nematode-infected roots. Instead, SA-associated changes in antioxidant capacity and redox signaling may contribute to HR, PR-gene expression, and systemic acquired resistance. In parallel, JA–ROS crosstalk acts in a context-dependent manner, contributing either to compatibility in susceptible hosts or to defense priming and PCD regulation in resistant hosts. Brassinosteroid (BR) signaling further enhances resistance through the RBOH1–MPK1/2–MPK3 pathway. (**B**) Systemic root-to-shoot signaling in tomato. RKN infestation induces GLR3.5-dependent electrical signaling together with an RBOH1-dependent ROS wave that propagates from infected roots to aerial tissues. These long-distance signals promote JA accumulation and MPK1/2 activation in shoots and contribute to systemic resistance against RKN infection. This tomato GLR3.5-dependent pathway is distinct from the GLR3.3/GLR3.6-mediated wound-signaling pathway characterized in *Arabidopsis*.

Collectively, ROS-dependent downstream defense in plant–RKN interactions is best understood as a multilayered network (Figure 5). Locally, H_2_O_2_ activates lignin, callose, and possibly suberin deposition while interacting with SA-dependent immunity through context-dependent changes in antioxidant capacity and redox signaling. Hormonal crosstalk further shapes the outcome: SA generally promotes resistance, JA can either support compatibility or defense depending on context, and BRs enhance RBOH1–MAPK-mediated resistance. Systemically, root-generated ROS and electrical signals coordinate JA accumulation and whole-plant resistance. Thus, ROS act not merely as local antimicrobial molecules but as integrative signals that connect root infection sites with structural, hormonal, and systemic immune programs.

## 8. Translational Redox Engineering for Durable Root-Knot Nematode Resistance

The growing mechanistic understanding of ROS regulation in RKN interactions provides several opportunities for crop improvement. Across multiple host species, resistance is commonly associated with early H_2_O_2_ accumulation, restrained H_2_O_2_ scavenging, activation of phenylpropanoid metabolism, and reinforcement of cell walls through lignin and callose deposition. These findings suggest that the plant redox network can be targeted through genome editing, transgenic or RNA interference (RNAi)-based approaches, and defense priming strategies. However, because excessive ROS can impair plant growth and cause oxidative injury, practical applications must aim to fine-tune ROS homeostasis rather than constitutively maximize ROS production.

### 8.1. Engineering Defense-Associated ROS Homeostasis

One promising strategy is to engineer crops to reproduce the redox signature observed in resistant cultivars: high or rapidly inducible ROS production combined with limited H_2_O_2_ scavenging during the early stages of nematode penetration. In principle, this could be achieved by enhancing root RBOH or SOD activity, suppressing selected CAT isoforms, or modifying regulatory elements controlling these genes. Overexpression of *RBOHD* or SOD in roots could sustain H_2_O_2_ accumulation at levels sufficient to activate HR-like defense, SA signaling, and phenylpropanoid metabolism. Conversely, targeted suppression or CRISPR/Cas9-mediated editing of root-expressed CAT isoforms could mimic the CAT-inhibited state observed in resistant tomato, sweetpotato, and carrot [14,45,46].

Nevertheless, constitutive enhancement of ROS production carries a risk of growth inhibition, premature senescence, or reduced yield under non-infected conditions. Therefore, root-specific, nematode-inducible, or chemically inducible promoters would be preferable to constitutive expression systems. Promoter editing of *RBOH* genes may also offer a more refined approach by increasing ROS production only in root tissues or only during early infection. Supporting this concept, CRISPR/Cas9-mediated mutation of the rice susceptibility gene *OsHPP04* increased apoplastic ROS accumulation, callose deposition, and resistance to the rice root-knot nematode without obvious developmental defects [4]. Similar editing of susceptibility genes such as *GmLMM1* in soybean, or orthologous ROS-regulatory susceptibility genes in other crops, may provide durable resistance without requiring continuous chemical inputs.

### 8.2. Targeting ROS-Suppressing Nematode Effectors

The discovery of nematode effectors that suppress host ROS signaling provides precise molecular targets for RNAi and related biotechnological approaches. Host-induced gene silencing (HIGS) can be used to express double-stranded RNA in the plant that targets essential nematode genes during parasitism. MjTTL5, a *M. javanica* effector that hijacks the host ferredoxin–thioredoxin system to enhance ROS scavenging, is a validated target: in planta RNAi-mediated silencing of *MjTTL5* reduced nematode reproduction by approximately 34–37.5% [7]. Similarly, CATLe, a catalase-like effector from *M. incognita* that directly decomposes H_2_O_2_, is highly suitable for HIGS because its silencing increases H_2_O_2_ accumulation at infection sites and reduces galling, gall size, and female development [76].

Stacking RNAi constructs that simultaneously target multiple ROS-suppressing effectors—such as *CATLe*, *MjTTL5*, and *Mi-CRT*—could provide stronger and more durable resistance than targeting a single effector. Nematode-intrinsic antioxidant genes, including PRXs, GSTs, SODs, and C-type lectins, also represent potential targets because they protect nematodes from host-derived ROS. RNAi-mediated knockdown of *M. incognita* PRXs has been reported to reduce root galling, indicating that nematode self-protection mechanisms are important for successful parasitism [5,8,78]. Although HIGS is powerful, its field deployment will require careful consideration of crop transformation efficiency, RNA stability, target specificity, regulatory approval, and durability against nematode sequence variation.

### 8.3. CRISPR/Cas-Based Editing of Host Susceptibility Pathways

Genome editing offers an alternative to transgenic expression of defense genes or dsRNA constructs. Rather than adding new resistance factors, CRISPR/Cas systems can disrupt or reprogram host susceptibility genes that are exploited by nematode effectors. For example, *OsHPP04* in rice acts as a susceptibility factor associated with suppression of ROS-mediated defense during *M. graminicola* infection; CRISPR/Cas9-edited *oshpp04* lines show higher ROS levels, stronger callose deposition, and enhanced nematode resistance [4]. This demonstrates that disabling host components used by nematodes to suppress ROS can convert a susceptible redox state into a resistant one.

Several candidate host targets emerge from current redox models. These include root-specific CAT isoforms that limit H_2_O_2_ accumulation, host targets of MjTTL5 such as ferredoxin–thioredoxin reductase components, extracellular Ca^2+^ signaling elements targeted by Mi-CRT, and regulatory regions of *RBOH* genes that control the timing and intensity of the oxidative burst. However, because these pathways also support normal development and stress tolerance, editing strategies should prioritize partial loss-of-function alleles, tissue-specific regulation, promoter editing, or inducible systems rather than complete disruption of essential redox genes.

### 8.4. Biocontrol-Mediated Priming of ROS-Associated Defense Against Root-Knot Nematodes

#### 8.4.1. Definition of Priming and Criteria for Evidence

Defense priming is a physiological state in which prior exposure to a biological or chemical stimulus enables a plant to activate defense responses more rapidly or more strongly upon subsequent challenge, without necessarily maintaining constitutively high defense activity [97]. This distinction is important in plant–RKN studies. An increase in antioxidant-enzyme activity or defense-gene expression after treatment does not, by itself, establish priming. Stronger evidence requires a treatment–challenge design showing that pretreated plants respond differently after nematode inoculation or a split-root design demonstrating that a locally applied microorganism induces resistance in a spatially separated root compartment.

Several studies now show that biological control agents can induce systemic resistance against RKNs and alter defense pathways associated with ROS metabolism. However, direct evidence that these treatments specifically prime an RBOH-dependent ROS burst remains limited. The available literature therefore provides stronger support for biological control agent (BCA)-mediated defense priming accompanied by redox-associated changes than for a defined, RBOH-dependent mechanism of ROS priming.

#### 8.4.2. Direct Evidence for Biocontrol-Mediated Priming and Systemic Resistance in Tomato

Molinari and Leonetti [98] provided direct evidence that pretreatment with beneficial biological control agents can prime susceptible tomato against *M. incognita*. Soil drenching with a mixture of biological control agents reduced nematode susceptibility and induced systemic expression of the SA-responsive marker *PR-1b* before nematode inoculation. Following inoculation, pretreated plants showed earlier or stronger expression of *PR-1*, *PR-3*, *PR-5*, and *ACC oxidase* genes than untreated infected plants. Glucanase and endochitinase activities were also enhanced in pretreated and inoculated roots. In addition, the marked infection-induced increase in *CAT* transcript abundance was suppressed or attenuated in BCA-treated roots.

These results are consistent with a primed immune state that includes altered antioxidant capacity. Nevertheless, the study did not directly measure H_2_O_2_ concentrations, ROS-production kinetics, or RBOH activity. The reduction in *CAT* transcript abundance should therefore be interpreted as evidence of altered redox regulation rather than as proof that BCA treatment generated a defined ROS burst or that SA directly inhibited CAT.

A complementary line of evidence was obtained using the endophytic bacterium *Bacillus cereus* BCM2. In a split-root system, bacterial inoculation of one root compartment reduced gall and egg-mass formation following *M. incognita* inoculation of a physically separated compartment [99]. RNA sequencing identified 34 candidate defense-related genes associated with BCM2-mediated resistance, and the plant–pathogen interaction pathway was significantly enriched. This experimental design provides strong evidence for systemic resistance and a priming-like response because the bacterium and nematode were spatially separated. However, the study did not establish that ROS constituted the mobile signal or that the induced resistance required RBOH activity.

More recent split-root experiments with *Bacillus velezensis* TA-1 further strengthened the connection between *Bacillus*-induced systemic resistance and redox-associated defense. Root colonization by TA-1 reduced gall formation in both locally treated and spatially separated root systems and was accompanied by increased H_2_O_2_ accumulation, lignin deposition, PAL, POD, and PPO activities, and expression of SA- and JA-responsive defense genes [100]. These findings demonstrate that systemic resistance induced by a *Bacillus* strain can be accompanied by enhanced oxidative and structural defense responses. However, they do not establish whether H_2_O_2_ accumulation was faster or stronger specifically because of priming, whether RBOH activity was required, or whether ROS were necessary for the protective effect.

#### 8.4.3. Redox-Associated Responses Induced by Individual *Bacillus* Strains

Other *Bacillus* studies support a connection between biological control and redox-associated defense, although they do not always distinguish host priming from direct nematicidal effects. Volatile compounds produced by *Bacillus atrophaeus* GBSC56 showed direct toxicity to *M. incognita* juveniles. In infected tomato roots, treatment with the bacterium or selected volatiles was also associated with increased SOD, CAT, POD, and APX activities and enhanced expression of defense-related genes, including *PR1*, *PR5*, and *SlLOX1* [101]. These observations demonstrate that bacterial volatiles can affect both the nematode and host defense physiology. However, increased antioxidant-enzyme activity may reflect enhanced detoxification of infection-associated oxidative stress and is not, by itself, evidence of a faster or stronger ROS burst.

*Bacillus velezensis* VB7 similarly suppressed *M. incognita* through a combination of direct nematicidal activity and induction of tomato defense responses [102]. Such dual activity is agronomically valuable, but it complicates mechanistic interpretation because reductions in penetration, galling, or reproduction cannot be attributed exclusively to host priming.

The effects of *Bacillus* are also strain-dependent. *B. velezensis* YS-AT-DS1 promoted tomato growth and reduced *M. incognita* infection, but split-root experiments did not support the induction of classical systemic resistance, and the tested SA- and JA-responsive genes were not activated [103]. Instead, the bacterium appeared to counteract nematode-associated changes in tonoplast intrinsic protein genes. This result demonstrates that successful biological control by a *Bacillus* strain does not necessarily involve classical ISR or ROS priming.

#### 8.4.4. Mycorrhiza-Induced Resistance and the Limits of Current Evidence

Arbuscular mycorrhizal fungi provide additional evidence that beneficial root-associated microorganisms can induce systemic resistance against nematodes. In tomato, colonization by *Glomus mosseae*, currently classified as *Funneliformis mosseae*, reduced infection by *M. incognita* in both local and split-root experiments [104]. The split-root results demonstrated that the protective effect was not restricted to the mycorrhiza-colonized root compartment and therefore supported mycorrhiza-induced systemic resistance.

However, this study did not directly measure ROS, RBOH activity, lignification, or callose deposition. It should therefore be cited as evidence of systemic resistance rather than as direct evidence of ROS priming. More broadly, studies of biological control agents often measure nematode reproduction, defense-related enzyme activities, or transcript abundance at only one or a few time points. These endpoints establish biocontrol efficacy and defense activation but rarely resolve whether ROS production is faster, stronger, or spatially redistributed in a primed plant.

Overall, the current evidence supports the conclusion that biological control agents can prime or induce systemic defense against RKNs and that these responses are frequently accompanied by changes in antioxidant metabolism, H_2_O_2_ accumulation, and defense-gene expression. Nevertheless, a direct causal role for RBOH-derived ROS in BCA-mediated priming remains to be demonstrated. Such a demonstration will require time-resolved analyses after nematode challenge, direct ROS imaging, appropriate untreated and unchallenged controls, and genetic or inducible manipulation of ROS-producing and ROS-scavenging systems.

### 8.5. Toward Integrated Redox-Based Nematode Management

The most durable application of ROS biology is likely to come from integrated strategies rather than single interventions. A practical redox-based management framework could combine resistant cultivars with genome-edited susceptibility genes, HIGS targeting conserved nematode ROS-suppressing effectors, and precisely timed chemical or biological priming. Such stacking would attack the compatibility process at multiple levels: increasing host ROS production, limiting host ROS scavenging, preventing nematode effector-mediated ROS suppression, and activating systemic defense before nematode establishment.

Future research should prioritize identifying infection-stage-specific promoters, validating root-specific CAT and RBOH isoforms across crop species, assessing yield penalties under field conditions, and testing whether combined strategies remain effective against genetically diverse Meloidogyne populations. Ultimately, sustainable nematode resistance will require redox engineering that maintains H_2_O_2_ within a defense-activating range during early infection while preserving normal growth and environmental resilience.

## 9. Conclusions and Future Perspectives: Redox Thresholds, Effector Arms Races, and Translational Opportunities

### 9.1. An Integrated Redox Model of Plant–RKN Interactions

Over the past two decades, ROS have emerged as central, bidirectional regulators of plant–RKN interactions. Rather than functioning solely as toxic antimicrobial molecules, ROS—particularly H_2_O_2_—act as dynamic signaling intermediates whose biological effects depend on their concentration, timing, subcellular origin, and spatial distribution. The evidence reviewed here supports a unified model in which the outcome of plant–RKN interactions is determined by the spatiotemporal H_2_O_2_ profile generated through RBOH-dependent production and reshaped by SOD-, CAT-, APX-, and POD-mediated scavenging [5,6,14].

In resistant interactions, nematode perception activates selected cell-surface receptor pathways, including some BAK1/SERK3-associated complexes, converge on BIK1, Ca^2+^ signaling, and RBOHD/F. This pathway generates an early oxidative burst that forms the first chemical defense layer against nematode invasion, and genetic disruption of BAK1, BIK1, RBOHD, or RBOHF increases susceptibility to *M. incognita* [5]. When this early burst is sustained and amplified through SA-associated immune signaling and reduced H_2_O_2_-scavenging capacity, H_2_O_2_ reaches defense-activating thresholds that trigger HR-associated cell death, phenylpropanoid metabolism, lignin and callose deposition, and restriction of feeding site establishment [6,14,45,46].

### 9.2. Conserved Resistance Signatures and Nematode Counter-Strategies

A major conclusion from comparative studies is that a recurring, but non-universal, pattern in several resistant plant–RKN systems is elevated SOD activity together with limited or unchanged H_2_O_2_-scavenging capacity. Elevated SOD activity channels superoxide radicals into H_2_O_2_, whereas suppressed or constrained CAT activity prevents rapid H_2_O_2_ degradation. This pattern has been observed across several plant systems, including tomato, sweetpotato, and carrot, and is linked to enhanced phenylpropanoid-mediated cell wall reinforcement rather than simple ROS scavenging [14,45,46]. Thus, the decisive factor is not total ROS production alone, but the plant’s ability to maintain H_2_O_2_ at the right level, time, and location to activate defense.

Conversely, compatible interactions are characterized by nematode-mediated redox manipulation. RKNs deploy diverse effectors that suppress ROS production, enhance ROS scavenging, or directly degrade H_2_O_2_. Mi-CRT may attenuate Ca^2+^-dependent immune signaling, although direct inhibition of RBOH activation has not been demonstrated. GrCEP12 suppresses upstream PTI outputs, and MiPDCD6 interferes with SA-mediated immunity. MjTTL5 exploits the host ferredoxin–thioredoxin system to enhance H_2_O_2_ scavenging, MgMO289 activates host superoxide-scavenging mechanisms, CATLe directly decomposes H_2_O_2_, and Mj-NEROSs reduces plastid-derived ROS by targeting electron transport components [7,8,41,43,76]. In addition, nematode-encoded antioxidant enzymes such as CATs, PRXs, SODs, and GSTs protect the parasite from host-derived oxidative stress [8]. These findings position ROS regulation as a major battleground in the coevolutionary arms race between plants and nematodes.

### 9.3. The ROS Paradox and the Importance of Redox Context

One of the most important conceptual advances in this field is the recognition that ROS are not exclusively defensive. RBOH-derived ROS can also promote compatible infection by restricting the spread of cell death around developing feeding sites. In the *Arabidopsis–Heterodera schachtii* system, *rbohD rbohF* mutants show reduced syncytium development but expanded cell death adjacent to infection sites, indicating that RBOH-derived ROS help maintain the living cellular environment required for nematode feeding [5,17]. Whether similar logic applies to RKN-induced giant cells remains unknown.

This paradox requires a shift from viewing ROS levels as simply “high” or “low” to considering ROS thresholds, localization, duration, and compartmentalization. Excessive H_2_O_2_ promotes HR, lignification, and resistance, whereas excessive ROS depletion may weaken basal defense or impair feeding site-associated developmental processes. Therefore, compatible parasitism may occur within an emergent redox range, but neither its quantitative boundaries nor a lower H_2_O_2_ requirement in RKN-induced giant cells has been established [17,18,76]. The Goldilocks framework should therefore be interpreted only as a working redox-threshold hypothesis.

### 9.4. Biocontrol-Mediated Priming: Established Evidence and Remaining Gaps

Biocontrol-mediated priming should no longer be regarded as an entirely unexplored aspect of plant–RKN interactions. Pretreatment–challenge and split-root experiments have demonstrated that mixtures of beneficial microorganisms, endophytic *Bacillus* strains, individual *Bacillus* isolates, and arbuscular mycorrhizal fungi can induce local or systemic resistance against RKNs. Several studies have also reported accompanying changes in H_2_O_2_ accumulation, antioxidant-enzyme activity, lignification, and defense-gene expression. However, the evidence for systemic resistance is currently stronger than the evidence for a specifically RBOH-dependent ROS-priming mechanism.

The major remaining questions are therefore mechanistic and translational. First, direct measurements are needed to determine whether BCA-treated plants produce ROS more rapidly, more strongly, or in different cell types after nematode challenge. Second, genetic or inducible manipulation of RBOH, CAT, APX, and other redox components is required to establish whether altered ROS metabolism is necessary for BCA-mediated resistance. Third, the direct nematicidal effects of microbial metabolites must be experimentally separated from host-mediated priming. Fourth, the active microbial elicitors and the extent of strain specificity remain incompletely defined. Fifth, the similarity between BCA-induced defense and genetic RKN resistance should be assessed using common temporal, cellular, and molecular endpoints. Finally, the persistence, reproducibility, and fitness costs of BCA-mediated priming must be evaluated under different soil types, microbiome compositions, climatic conditions, and nematode pressures. Evidence for transgenerational inheritance of BCA-induced ROS priming against RKNs remains insufficient and should be treated as a future research question rather than as an established mechanism.

### 9.5. Translational Outlook

The convergence of ROS biology with CRISPR/Cas genome editing, host-induced gene silencing, chemical priming, and precision biocontrol creates a promising framework for sustainable nematode management. Future resistance strategies should not aim to maximize ROS constitutively; instead, they should engineer infection-stage-specific redox responses that elevate H_2_O_2_ during early nematode invasion while preserving normal growth and stress tolerance.

Durable resistance will likely require stacked strategies that combine CRISPR-engineered host susceptibility genes, optimized SOD–CAT balance, RNAi targeting of conserved nematode ROS-suppressing effectors, and chemical or microbial priming of PTI and SA-dependent defenses. Such integrated approaches could reduce reliance on chemical nematicides and provide environmentally sustainable protection for economically important crops. Ultimately, the central challenge is to transform mechanistic knowledge of redox dynamics into field-stable resistance systems that maintain H_2_O_2_ within a defense-activating but developmentally tolerable range.

## Figures and Tables

**Table 1:** Nematode effectors and antioxidant systems targeting host ROS networks during interactions with root-knot and related sedentary plant-parasitic nematodes.

Effector or System	Nematode Species	Main Host Target or Pathway	Redox-Related Mechanism	Biological Outcome	References
Mi-CRT	*M. incognita*	Apoplastic Ca^2+^ signaling; RBOHD activation	Binds apoplastic Ca^2+^ and may attenuate Ca^2+^-dependent immune signaling; direct inhibition of RBOHD activation has not been demonstrated.	Reduces ROS production, defense gene expression, and callose deposition; promotes early immune suppression and nematode infection	[20,27,72]
GrCEP12	*G. rostochiensis*	PTI signaling upstream of RBOH	Suppresses flg22-induced PTI outputs, including ROS burst, MAPK activation, callose deposition, and PTI marker gene expression	Weakens upstream immune signaling and reduces RBOH-mediated ROS production	[7,73,74]
MiPDCD6	*M. incognita*	SA-mediated immunity	Suppresses SA-dependent defense responses, indirectly reducing defense-associated ROS accumulation	Promotes compatible interaction by weakening SA-associated redox defense	[43,75,76]
MgMO289	*M. graminicola*	Rice OsHPP04-associated O_2_•^−^ scavenging pathway	Interacts with OsHPP04, increases host cytosolic Cu/Zn-SOD2 activity, and lowers O_2_•^−^ availability.	Lowers superoxide availability and contributes to suppression of ROS-mediated defense	[64]
MjTTL5	*M. javanica*	Plastid ferredoxin–thioredoxin reductase system; AtFTRc	Localizes to plastids and interacts with AtFTRc, enhancing host H_2_O_2_-scavenging capacity	Suppresses ROS-mediated immunity; transgenic expression increases susceptibility, whereas RNAi-mediated silencing reduces nematode reproduction by approximately 34–38%	[7,18,43]
CATLe	*M. incognita*	H_2_O_2_ at the plant–nematode interface	Functions as a catalase-like effector that directly decomposes H_2_O_2_; possesses strong catalase activity	Dampens PTI-associated ROS; CATLe silencing increases H_2_O_2_ and reduces gall number, gall size, and egg mass production	[43,76]
Mj-NEROSs	*M. javanica*	Plastid Rieske iron–sulfur protein; cytochrome *b_6_f* complex	Alters plastid electron transport and reduces plastid-derived ROS production	Suppresses flg22-induced ROS, INF1-triggered cell death, and callose deposition; weakens plastid-based defense	[28,43]
CMII	*M. incognita*	Chorismate metabolism; SA biosynthesis	Diverts chorismate toward prephenate metabolism, thereby attenuating chorismate-dependent SA biosynthesis and SA-associated defense. Downstream effects on CAT, APX, and H_2_O_2_ homeostasis remain indirect and context-dependent.	Weakens SA-associated immunity and may shift host redox conditions toward compatibility.	[43,77]
Peroxiredoxins	*M. incognita*	Nematode-intrinsic antioxidant defense	Reduce H_2_O_2_ using thioredoxin and detoxify plant-derived apoplastic ROS near the nematode cuticle and hypodermis	Protect nematodes from host oxidative stress; RNAi knockdown reduces root galling	[78]
Nematode antioxidant and ROS-modulatory proteins	*M. incognita* and other RKNs	Nematode self-protection machinery	Catalase, SOD, GPX, GSTs, peroxiredoxins, and C-type lectins detoxify ROS or suppress host ROS production	Enable survival under elevated host ROS, especially during resistant interactions; may reflect stress responses to failed feeding site maintenance	[8,62,64,75,77]
Combined ROS-targeting effector network	RKNs and related sedentary nematodes	PTI signaling, RBOH activation, plastid redox metabolism, H_2_O_2_ detoxification, and SA-dependent defense	Multiple effectors act at distinct redox nodes to attenuate, redistribute, or buffer ROS rather than eliminate ROS completely	May shift host redox conditions toward compatibility through combined attenuation of ROS production and enhancement of ROS removal; active maintenance of a fixed H_2_O_2_ setpoint has not been demonstrated.	[5,7,8,16,18,43,71]

## Data Availability

No new data were created or analyzed in this study.

## Abbreviations

ABA	Abscisic acid
ACO	1-Aminocyclopropane-1-carboxylate oxidase
APX	Ascorbate peroxidase
AsA	Ascorbate/Ascorbic acid
BAK1	Brassinosteroid insensitive 1-associated receptor kinase 1
BCA	Biological control agent
BIK1	Botrytis-induced kinase 1
BR	Brassinosteroid
CAT	Catalase
CATLe	Catalase-like effector
CeCl_3_	Cerium chloride
CERK1	Chitin elicitor receptor kinase 1
CMII	Chorismate mutase II
CRISPR/Cas	Clustered regularly interspaced short palindromic repeats/CRISPR-associated system
DAMP	Damage-associated molecular pattern
DCFH	2′,7′-Dichlorodihydrofluorescein
DHAR	Dehydroascorbate reductase
DIECA	Sodium diethyldithiocarbamate
DPI	Diphenyleneiodonium
DMTU	Dimethylthiourea
DORN1	Does not respond to nucleotides 1
dsRNA	Double-stranded RNA
ET	Ethylene
ETI	Effector-triggered immunity
FLS2	Flagellin-sensitive 2
FTR	Ferredoxin–thioredoxin reductase
G6PDH	Glucose-6-phosphate dehydrogenase
GLR	Glutamate receptor-like channel
GPX	Glutathione peroxidase
GR	Glutathione reductase
GSH	Reduced glutathione/Glutathione
GmLMM1	Glycine max lesion mimic mutant 1
GST	Glutathione S-transferase
HIGS	Host-induced gene silencing
HR	Hypersensitive response
HY5	Elongated hypocotyl 5
IEF	Isoelectric focusing
ISP	Iron–sulfur protein
J2	Second-stage juvenile
JA	Jasmonic acid
JERF3	Jasmonate/ethylene-responsive factor 3
LRR	Leucine-rich repeat
LYK5	LysM domain-containing receptor-like kinase 5
MAMP	Microbe-associated molecular pattern
MAPK	Mitogen-activated protein kinase
Mi-CRT	*Meloidogyne incognita* calreticulin
MiPDCD6	*Meloidogyne incognita* programmed cell death protein 6-like effector
Mj-NEROSs	*Meloidogyne javanica* nematode effector ROS suppressor
MjTTL5	*Meloidogyne javanica* transthyretin-like protein 5
MgMO289	*Meloidogyne graminicola* effector MO289
MPK	Mitogen-activated protein kinase
NADH	Nicotinamide adenine dinucleotide, reduced form
NADPH	Nicotinamide adenine dinucleotide phosphate, reduced form
NAMP	Nematode-associated molecular pattern
NBS	Nucleotide-binding site
NBT	Nitro blue tetrazolium
NLR	Nucleotide-binding leucine-rich repeat receptor
NPR1	Nonexpressor of pathogenesis-related genes 1
OPPP	Oxidative pentose phosphate pathway
PAMP	Pathogen-associated molecular pattern
PAL	Phenylalanine ammonia-lyase
PCD	Programmed cell death
PEPR	PEP receptor
POD	Peroxidase
PP2C	Protein phosphatase 2C
PR	Pathogenesis-related
PRR	Pattern recognition receptor
PRX	Peroxiredoxin
PTI	Pattern-triggered immunity
RBOH	Respiratory burst oxidase homolog
RKN	Root-knot nematode
RLK	Receptor-like kinase
RNAi	RNA interference
ROS	Reactive oxygen species
SA	Salicylic acid
SAR	Systemic acquired resistance
SERK3	Somatic embryogenesis receptor-like kinase 3
SHAM	Salicylhydroxamic acid
SOD	Superoxide dismutase
TGA	TGACG-binding transcription factor
TRX	Thioredoxin
WAT	Walls are thin
WFI	Wound-induced protein
